# Qualitative Studies of Infant and Young Child Feeding in Lower-Income Countries: A Systematic Review and Synthesis of Dietary Patterns

**DOI:** 10.3390/nu9101140

**Published:** 2017-10-18

**Authors:** Alessandra N. Bazzano, Aiko Kaji, Erica Felker-Kantor, Lydia A. Bazzano, Kaitlin S. Potts

**Affiliations:** 1 Department of Global Community Health and Behavioral Sciences, Tulane University School of Public Health and Tropical Medicine, New Orleans, LA 70112, USA; akaji@tulane.edu (A.K.); efelkerk@tulane.edu (E.F.-K.); kstorck@tulane.edu (K.S.P.); 2 Department of Epidemiology, Tulane University School of Public Health and Tropical Medicine, New Orleans, LA 70112, USA; lbazzano@tulane.edu

**Keywords:** qualitative research, infant nutritional physiological phenomena, breast feeding, feeding behavior

## Abstract

Continued high rates of both under- and over-nutrition in low- and low-middle-income countries highlight the importance of understanding dietary practices such as early and exclusive breastfeeding, and dietary patterns such as timely, appropriate complementary feeding—these behaviors are rooted in complex cultural ecologies. A systematic review and synthesis of available qualitative research related to infant and young child dietary patterns and practices from the perspective of parents and families in low income settings is presented, with a focus on barriers and facilitators to achieving international recommendations. Data from both published and grey literature from 2006 to 2016 was included in the review. Quality assessment consisted of two phases (Critical Appraisal Skills Program (CASP) guidelines and assessment using GRADE-CERQual), followed by synthesis of the studies identified, and subsequent thematic analysis and interpretation. The findings indicated several categories of both barriers and facilitators, spanning individual and system level factors. The review informs efforts aimed at improving child health and nutrition, and represents the first such comprehensive review of the qualitative literature, uniquely suited to understanding complex behaviors leading to infant and young child dietary patterns.

## 1. Introduction

Early nutrition is crucially important for children to survive, grow and develop into healthy adults who can lead rewarding lives and productively contribute to their communities, and improving nutrition among young children has widely been recognized as an international priority [1]. Nonetheless, almost half of all childhood deaths (those under age 5) continue to be linked to nutritional causes [2]. In addition to morbidity and mortality, the impacts of poor nutrition, both in the form of under- and overnutrition, include strong, negative intergenerational health consequences for descendants [3]. Alongside maternal nutrition and intrauterine exposures [4], nutritionally-based caring behaviors of families, including breastfeeding and complementary feeding, form the basis of child nutrition. As a result, the potential impact of improved dietary practices on child nutrition has been extensively studied and subsequently promoted for potential to greatly improve health [5,6,7,8].

The United Nations Children’s Fund (UNICEF) and the World Health Organization (WHO) concur that optimal nutrition practices for childhood include early initiation of breastfeeding, exclusive breastfeeding for the first 6 months of life, followed by the addition of nutritionally adequate, safe, and appropriate complementary foods with continuation of breastfeeding for 1 year and longer [9,10]. Markers of appropriate infant and young child feeding include these practices, as well as the timely introduction of solid and semi-solid foods, adequately frequent provision of daily meals, dietary diversity, and consumption of iron rich foods. In response to the need for simple, practical indicators of appropriate feeding practices in children aged 6–23 months, WHO published a set of population-level indicators that could be obtained from large-scale survey data on feeding practices in varied international settings [11].

However, characterizing feeding practices through quantitative methods such as large-scale surveys can be challenging, particularly because these practices constitute multidimensional and often interrelated behaviors rooted in family systems and socioeconomic conditions, and feeding patterns also change rapidly within short intervals of age [12,13]. Nonetheless, information on infant and young child feeding (IYCF) practices is required to improve nutrition and health during the first 2 years of life. Over the last decade, numerous studies on improvement of nutrition-related behaviors for infants and young children have relied on qualitative research methods, well-suited to exploring complex behaviors and their underlying psychosocial and cultural drivers, to investigate infant and young child feeding [14,15,16,17,18]. This body of research is likely to contain useful information to further the understanding of behavioral approaches to improve child nutrition. A comprehensive summary of qualitative data on feeding practices in this age group from lower-income settings, including a synthesis of barriers and facilitators to recommended practices, is currently lacking in the biomedical literature.

The primary objective of the proposed study was to systematically review qualitative literature related to family experiences (particularly parental ones) of infant and young child feeding in low-income countries, synthesizing information on the barriers and facilitators that may relate to interventions to impact nutrition, survival, growth and development. The results provide an overview of qualitative studies relating parental perspectives on infant and child dietary patterns, in the interest of providing insights for developing, improving, and scaling nutrition interventions.

## 2. Materials and Methods

This systematic review was registered with the International Prospective Register of Systematic Reviews (PROSPERO): registration number CRD42016035677. The review followed guidelines from the Enhancing Transparency in Reporting the Synthesis of Qualitative Research (ENTREQ) statement. Due to the exclusive focus on qualitative research, the review employed the ENTREQ guidelines [19] in lieu of the Preferred Reporting Items for Systematic Reviews and Meta-Analyses guidelines, which are more specific to the requirements of quantitative literature reviews [20]. The Supplementary Material to this article contains the full ENTREQ checklist.

Studies were only reviewed if their results were directly obtained from participants who were parents or close family members of an infant or young child (0–2 years of age) at the time of the study. Family members, for the purposes of this review, were defined to include mothers, fathers or other caregivers living in the child’s household who routinely engage in infant or young child feeding. Infant and child feeding practices were defined as all actions taken to meet the physiological nutritional needs of children in this age group, including but not limited to: breast feeding; introduction of solid, semi-solid, and/or family foods (known as complementary feeding); and continued breastfeeding of children alongside provision of solid/semi-solid food.

### 2.1. Inclusion and Exclusion Criteria

Included studies used widely accepted qualitative data collection methods, with well-described methodology, including for example: interviews, focus groups, direct observation, and participatory action research. Included studies also needed to have provided a clear description of recognized qualitative data analysis methods (e.g., Grounded Theory, narrative analysis, content analysis, thematic analysis).

Excluded studies were those for which it was difficult to extract qualitative data, e.g., mixed methods studies without clearly labeled data, or studies in settings where perceptions of parents or caregivers around infant and young child feeding could not be clearly identified, such as summaries or aggregated data. Commentaries, protocols, and systematic reviews were not included in the analysis. Additionally, as the focus was on research from resource limited settings, studies from countries other than those defined by the World Bank as low-income countries and lower-middle income countries (which have a Gross National Income per capita of less than $4125) were excluded [21].

### 2.2. Search Strategy

The following electronic databases were considered to be the most relevant for the topic and were searched: MEDLINE (PubMed); Embase; Cumulative Index to Nursing and Allied Health Literature (CINAHL: EBSCOhost). A health sciences librarian was consulted in the development of the database searching strategy.

The initial search strategy was developed for MEDLINE and then adapted for the other databases. Medical Subject Headings were initially used followed by free-text terms using controlled vocabulary (see Appendix A for a detailed description of the search strategy). Results were restricted to English language publications from the last 10 years, due to potential difficulties in translating and interpreting foreign language qualitative data by native English-speaking reviewers, and to ensure that the review identifies literature relating to the most current infant and young child feeding practices. In addition, reference lists of included studies were manually searched to identify any additional studies that fit the inclusion criteria.

The included grey literature was initially identified through listing of relevant websites to search for organizations working in nutrition in lower-income countries (in consultation with experts working in the field who use and disseminate data through websites for related nutrition research). A custom search engine was created using Google Custom Search. Within the Custom Search Engine, the relevant websites were searched using search strategies adapted from those used in the databases to reflect relevant keywords related to qualitative studies of infant and young child feeding practices.

The review also included documents identified through a manual review of organizational reports and reports from relevant meetings related to nutrition of young children in low income countries. Results were similarly limited to publications in English from the last 10 years.

**Figure 1:**
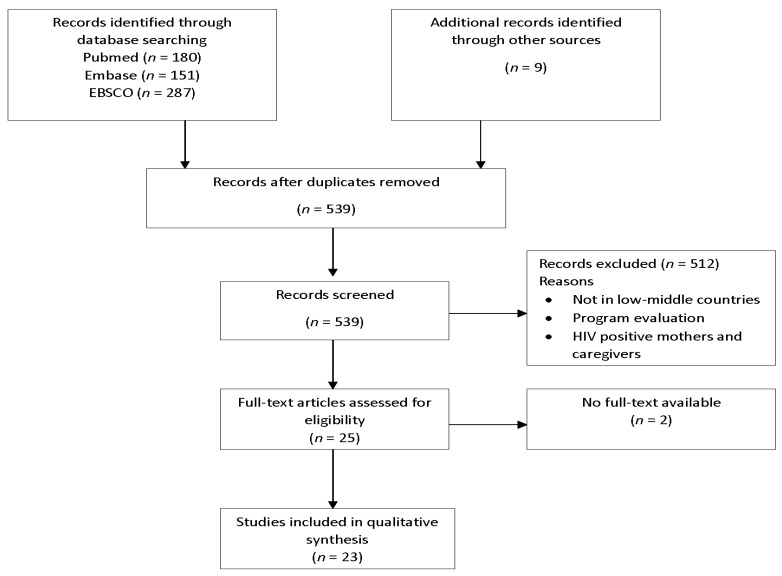
Flow diagram of search results and selection process.

A flow diagram using PRISMA guidelines for reporting of systematic reviews is presented in Figure 1 in reporting of the selection process and results [20]. For organization of initial search results, Endnote reference management software (EndNoteX8, Clarivate Analytics, Boston, MA, USA) was used, and results of searches imported to the software. At the first stage, duplicates and irrelevant studies were removed. Two independent reviewers then screened study titles and abstracts for suitability against inclusion and exclusion criteria. The decision to include or exclude a study was required to be agreed on by both reviewers. If after consultation a decision was not reached by the two reviewers, a third reviewer made the final decision.

### 2.3. Data Extraction

For organization of extracted data, a unified matrix was utilized to record specific characteristics of included studies. Extracted data included: reference details (author, year, title, journal/publisher); country/region of study; objectives or aims of the study; study design including methodological approaches (e.g., interviews/focus groups) and conceptual basis underlying the study (e.g., Grounded Theory); analysis method(s); sampling methodology and sample size; and initial assessment of the methodological limitations of the study. The initial results of the selection process and data abstraction are presented in Table 1.

Additional steps were taken in the data extraction phase that involved expanding the matrix (Table 1) to include participant characteristics, summaries of key outcomes/results reported, and the emergent review findings for which the study contributes evidence. These details are given in Appendix B.

### 2.4. Quality Appraisal

Each selected document was initially assessed for quality and internal validity according to the Critical Appraisal Skills Program (CASP) checklist for qualitative research [22]. The CASP checklist includes 10 questions to appraise the quality of qualitative research. These assessments for each study can be seen in the final column of Table 1 with reference to the CASP appraisal question number where the study presented potential quality limitations. Selected studies met minimum criteria defined through the checklist including domains such as appropriateness of study design, data collection techniques, and analysis methods used. CASP questions include the following:

1. Was there a clear statement of the aims of the research?
2. Is a qualitative methodology appropriate?
3. Was the research design appropriate to address the aims of the research?
4. Was the recruitment strategy appropriate to the aims of the research?
5. Was the data collected in a way that addressed the research issue?
6. Has the relationship between researcher and participants been adequately considered?
7. Have critical issues been taken into consideration?
8. Was the data analysis sufficiently rigorous?
9. Is there a clear statement of findings?
10. How valuable is the research?

At the second level of appraisal, the GRADE-CERQual (Grade Conidence Evidencerom Reviews Qualitative research) guidance [23] was used to differentiate emergent findings strongly supported or less well supported. Two reviewers independently reviewed studies. Reviewers used guidance derived from GRADE-CERQual to reach consensus of the quality of findings emergent from included studies. The results of this appraisal are presented in Table 2. During this process, methodological limitations of emergent findings were considered and categorized as minor, moderate or major. These categorizations were based on the presence or absence of description of key elements in methodology (guided by the CASP assessment in Table 1) in the document(s) which support the finding, including the following: approach used to recruit or sample participants, how potential for researcher bias was addressed, how analysis was done or, in the case of mixed methods research, how statistical sampling and analysis was done. Minor methodological limitations were those with one element missing, moderate limitations were those with two elements missing, and major limitations were those with three or more missing. Full details of which elements were missing from each document are available in Appendix C. Additional assessments of emergent findings were conducted based on the quality of the studies or documents supporting the finding. These included assessments of relevance, coherence and adequacy guided by the GRADE CERQual methodology. Assessments of relevance and coherence were not conducted for emergent findings with minimal (usually one) studies or grey literature contributing to the finding. These assessments for each emergent finding are given in Table 2 with full details of the how assessments were made found in Appendix C. The overall assessment of confidence in emergent findings was based upon the assessments of methodological limitations, relevance, coherence and adequacy and was guided by the GRADE CERQual method. These are given for each emergent finding in Table 2 along with an explanation of judgment describing how the level of confidence was reached.

Following abstraction and grading of evidence, the final step involved data from the results, discussion, and conclusion sections of the included studies, being extracted and entered into NVivo 11 qualitative software (QSR International Pty Ltd., Version 11, 2015, Burlington, MA, USA), wherein thematic analysis was employed to identify domains descriptive of the data for investigation and presentation.

This thematic analysis led to a more interpretive phase in order to understand how the themes identified may represent barriers and facilitators to change infant and young child feeding; for this we used an approach similar to that of Thomas et al. [24].

## 3. Results

After a narrative summary of themes identified through the initial analysis was available, three reviewers met jointly to consider these and produce a consensus-based listing of barriers and facilitators. This step evaluated all emergent findings in light of the summary of themes wherein several emergent findings were often merged into one synthesized finding, or individual emergent findings were discarded due to weak assessments of confidence or minimal support from the included studies. Table 3 presents the synthesized review findings of barriers and facilitators generated through the data synthesis exercise and subsequently agreed on by reviewers.

Four categories of barriers to recommended breastfeeding practices were identified, and three categories of barriers to recommended complementary feeding practices were identified. None of the studies included in the final review disclosed funding from food manufacturers, nor disclosed any related conflicts of interests. Barriers to breastfeeding included factors specific to infant or mother, and cross-cutting beliefs and perceptions, as well as a pervasive lack of support for breastfeeding, from families, health workers and due to time poverty. Several categories of facilitators were also identified from the literature reviewed, including food security, social support, and individual infant and maternal factors.

## 4. Conclusions

The findings contribute to the expanding literature on family experiences related to breastfeeding and complementary feeding of young children and infants in low-income settings and constitute the most comprehensive summary of findings to date. Previous systematic reviews of qualitative literature related to infant feeding have not included as broad an approach to the topic as the current review, having focused rather on specific areas such as bottle feeding [25] and obesogenic dietary intake. [26] Another qualitative review, undertaken in 2008, focused solely on maternal support for breastfeeding mothers [27] and one carried out in 2013 considered the psychosocial correlates of exclusive breastfeeding [28]. Neither of those included a comprehensive approach to assessing the quality of included studies.

Strengths of the current review included the use of multiple reviewers experienced in qualitative research and data collection and analysis, a comprehensive search strategy, assessment and scoring of quality and confidence placed in the findings based on guidelines, and inclusion of grey literature. Limitations of the study were the exclusion of documents not available in the English language and date limitations. Two authors of the present review, who conduct research in this topical area, have authored or co-authored articles included in the review, which inclusion was based solely on having met the eligibility criteria.

Overall, this review focused on identifying qualitative and ethnographic studies that related the experiences and first-hand accounts of family members responsible for providing for the care and nutritional needs of young children under 2 years of age. Through this systematic qualitative review and synthesis, hypothesized barriers and facilitators to improving infant and young child feeding have been identified. The findings presented in this review are directly applicable to social and behavioral change initiatives in low resource settings aimed at improving dietary patterns and practices for better health and nutrition of young children.

## Figures and Tables

**Table 1:** Data extraction of studies included in review including bibliographic information, study details and assessment of methodological limitations.

No.	Author, Year, and Country of Study	Title	Source Type	Aims of the Study	Study Design, Analysis Method (AM)	Sampling Method, Sample Size	Assessment of Methodological Limitations of Study (Numbers Refer to CASP Questions)
*Studies Identified from Search Databases*							
1	LM Babington 2006 Dominican Republic	Understanding Beliefs, Knowledge, and Practices of Mothers in the Dominican Republic Related to Feeding Infants and Young Children	Journal: Hispanic Health Care International	To expand knowledge regarding feeding practices and nutritional beliefs of mothers living in the Dominican Republic.	Qualitative study (focus group discussion) AM: Modified constant comparative method	Convenience sampling, 10 mothers aged 16–45 years with at least one child	5. no discussion of data adequacy/saturation; limited participants; limited explanation of results; few illustrative quotes 6. no discussion about the researcher’s role, potential bias and influence 8. limited data to support the findings
2	A Omer-Salim, Persson, P Olsson 2007 Tanzania	Whom can I rely on? Mothers’ approaches to support for feeding: An interview study in suburban Dar es Salaam, Tanzania	Journal: Midwifery	To explore and describe mothers’ perception of baby feeding and approaches to support for baby feeding & to describe and explore mothers’ experiences of support for baby feeding, with a focus on the transition from exclusive to non-exclusive breast feeding, in suburban Dar es Salaam, Tanzania.	Qualitative study (semi-structured interviews) AM: Qualitative content analysis	Purposive sampling from a clinic, 8 mothers with babies under 6 months of age	5. no discussion regarding saturation of data; limited participants 6. no discussion about the researcher’s role, potential bias and influence
3	C Tawiah-Agyemang, BR Kirkwood, K Edmond, A Bazzano, Z Hill 2008 Ghana	Early initiation of breast-feeding in Ghana: barriers and facilitators	Journal: Journal of Perinatology	To explore why women in Ghana initiate breast-feeding early or late, who gives advice about initiation, and what foods or fluids are given to babies when breast-feeding initiation is late.	Qualitative study AM: Thematic analysis	Purposive sampling, 52 semi-structured interviews with recently delivered mothers 8 focus group discussions (FGD) with women of child-bearing age	6. no discussion about the researcher’s role, potential bias and influence 7. no explicit information about approval from an ethics committee
4	GE Otoo, A. Lartey, R Pérez-Escamilla 2009 Ghana	Perceived Incentives and Barriers to Exclusive Breastfeeding Among Peri-urban Ghanaian Women	Journal: Journal of Human Lactation	To explore Ghanaian women’s knowledge and attitudes toward exclusive breastfeeding	Qualitative study (focus group discussions) AM: Thematic analysis	Convenience sampling, 4 focus group discussions with 7 to 10 mothers who had at least one child less than 4 months	6. no discussion about the researcher’s role, potential bias and influence
5	L Hernandez, ML Vasquez 2010 Colombia	Practices and beliefs about exclusive breastfeeding by women living in Commune 5 in Cali, Colombia	Journal: Colombia Médica	To describe the practices and beliefs about breastfeeding during the first 6 months after delivery	Qualitative study (Ethnonursing) AM: Spradley’s method; (identification of domains, taxonomies, componential analysis, and finally themes)	Purposive sampling, 15 key informant participants (three interviews for each participant: 45 interviews total); other participants (relatives of the key informant)	6. no discussion about the researcher’s role, potential bias and influence 7. no explicit information about approval from an ethics committee
6	S Rasheed, R Haider, N Hassan, H Pachon, S Islam, CS. Jalal, TG Sanghvi 2011 Bangladesh	Why does nutrition deteriorate rapidly among children under 2 years of age? Using qualitative methods to understand community perspectives on complementary feeding practices in Bangladesh	Journal: Food and Nutrition Bulletin	To understand community perspectives on complementary feeding practices in order to inform the design of future interventions for improved complementary feeding	Mixed method study (semi-structured interviews, food attributes exercises, opportunistic observations, trials of improved practices, key informant interviews, focus group discussions) AM: Thematic analysis; data triangulation of different data sources identified themes to understand phenomena	Opportunistic sampling, 42 semi-structured interviews with mothers; 6 interviews with key informants; 4 focus group discussions with 2 grandmothers and fathers; food attribute exercises with 24 mothers; and 21 opportunistic observations of feeding times involving meals or snacks	6. no discussion about the researcher’s role, potential bias and influence
7	PC Lundberg, TT Ngoc Thu 2011 Vietnam	Breast-feeding attitudes and practices among Vietnamese mothers in Ho Chi Minh City	Journal: Midwifery	To describe breast-feeding attitudes and practices among Vietnamese women in Ho Chi Minh City.	Qualitative study (semi-structured, in-depth interviews and observation) AM: Qualitative content analysis	Purposive sampling, 23 mothers who had given birth to a child within the last two years	6. no discussion about the researcher’s role, potential bias and influence
8	Y Afiyanti, D Juliastuti 2012 Indonesia	Exclusive breastfeeding practice in Indonesia	Journal: Birth Journal of Midwifery	To explore cultural practices and behaviors of breastfeeding among Indonesian mothers	Qualitative study (semi-structured interviews) AM: Grounded Theory, Constant comparative analysis	Purposive and theoretical sampling, 8 key participants (breastfeeding mothers) 10 general participants (family members and healthcare cadre)	5. no explanation regarding the approach for research 6. no discussion about the researcher’s role, potential bias and influence
9	HMH Lee, J Durham, J Booth, V Sychareun 2013 Lao PDR	A qualitative study on the breastfeeding experiences of first-time mothers in Vientiane, Lao PDR	Journal: BMC Pregnancy and Childbirth	To identify the reasons and influences for feeding decisions of first-time mothers in Lao PDR. Focusing on decisions around when to initiate breast feeding, timing of the introduction of complementary foods and breast feeding duration.	Qualitative study (interviews and focus group discussions) AM: Thematic analysis	Purposive sampling, In-depth interviews with16 first-time mothers and 7 key informants; focus group discussions with 24 first-time mothers	6. no discussion about the researcher’s role, potential bias and influence
10	C Bomer-Norton 2013 Haiti	Timing of breastfeeding initiation in rural Haiti: A focused ethnography	Dissertation: The University of Texas at Arlington	To describe factors affecting the timing of breastfeeding initiation among Haitian mothers, particularly the specific cultural beliefs and practices	Qualitative study (ethnography) AM: Ethnography	Purposive sampling, 25 mothers with children less than 12 months old	6. no discussion about the researcher’s role, potential bias and influence
11	A Laterra, MA Ayoya, JM Beauliere, M Bienfait, H Pachon 2014 Haiti	Infant and young child feeding in four departments in Haiti; mixed-method study on prevalence of recommended practices and related attitudes, beliefs, and other determinants	Journal: Pan American Journal of Public Health	To determine and describe the prevalence and patterns of three recommended practices for infant and young child feeding—exclusive breastfeeding, continued breastfeeding, and achievement of minimum dietary diversity To identify the attitudes and beliefs that inform these practices and any other factors that may facilitate or impede their implementation	Mixed method study (focus group discussions) AM: Thematic content analysis	Convenience sampling of survey respondents, 12 focus group discussions among women ≥18 years old with children ≤ 2 years old	5. no information regarding the format of data collection (e.g., tape recordings, notes etc.); no discussion about saturation of data 6. no discussion about the researcher’s role, potential bias and influence
12	G Pelto, M Armar-Klemesu 2015 Kenya	Identifying interventions to help rural Kenyan mothers cope with food insecurity: results of a focused ethnographic study	Journal: Maternal and Child Nutrition	To identify potential interventions to improve the quality, availability, and affordability of foods consumed by infants and young children	Qualitative study (semi-structured interviews, community observations, key informants). AM: Focused ethnographic study	Random selection of 32 caregivers, 8–10 key informants (no description of how informants were selected).	5. no explanation regarding the sampling or collection format of data; no discussion about saturation of data 6. no discussion about the researcher’s role, potential bias and influence
13	AN Bazzano, RA Oberhelman, KS Potts, LD Taub, C Var 2015 Cambodia	What health service support do families need for optimal breastfeeding? An in-depth exploration of young infant feeding practices in Cambodia	Journal: International Journal of Women’s Health	To provide an in-depth understanding of breastfeeding practices in order to appropriately design a scalable newborn health intervention incorporating expanded breastfeeding counseling and support	Qualitative study (semi-structured interviews with caregivers & healthcare providers) AM: Formative research for intervention development	Purposive sampling, 27 interviews, 14 observations	5. no explanation regarding the approach for research; no discussion about saturation of data 6. no discussion about the researcher’s role, potential bias and influence
14	J Burns, JA Emerson, K Amundson, S Doocy, LE Caulfield, RD Klemm 2016 Democratic Republic of Congo	A Qualitative Analysis of Barriers and Facilitators to Optimal Breastfeeding and Complementary Feeding Practices in South Kivu, Democratic Republic of Congo	Journal: Food and Nutrition Bulletin	To characterize IYCF practices and barriers and enablers to optimal child feeding in South Kivu	Qualitative study (focus group discussions, structured and in-depth interviews) AM: Thematic analysis	Convenience sampling, 188 participants in 20 focus group discussions (mothers of children < 24 months of age and women of reproductive age), 119 structured interviews (mothers with children < 24 months of age), 43 in-depth interviews (lead mothers).	6. no discussion about the researcher’s role, potential bias and influence
*Grey Literature*							
15	Alive and Thrive-Ethiopia 2010 Ethiopia	IYCF Practices, Beliefs, and Influences in Tigray Region, Ethiopia	Alive and Thrive	To understand infant and young child feeding practices and the role of service providers in the study communities.	Qualitative study (focus group discussion, interviews, observations) AM: Interpretative approach that involved eliciting meanings from the collected information	Random selection of respondents from the health post, and door to door sampling of households with children in the required age group; 45 interviews; FGD (grandmothers and fathers)	6. no discussion about the researcher’s role, potential bias and influence 8. lack of an in-depth description of the analysis process
16	Alive and Thrive 2010 Ethiopia	IYCF Practices, Beliefs, and Influences in SNNP Region, Ethiopia	Alive and Thrive	To understand infant and young child feeding practices and the role of service providers in the study communities	Qualitative study (focus group discussion, interviews, observations) AM: Interpretative approach that involved eliciting meanings from the collected information	Respondents were randomly selected in each area from a list provided by the health post, and the study interviewers also went door to door to identify households with children in the required age group; 47 interviews (mothers); FGD (grandmothers and fathers)	6. no discussion about the researcher’s role, potential bias and influence 8. lack of an in-depth description of the analysis process
17	United States Agency for International Development 2010	Formative assessment of infant and young child feeding practices at the community level in Zambia	United States Agency for International Development	The objectives of this study were to: describe the key influencers of infant and young child feeding practices in targeted areas in Zambia; examine the barriers and constraints to the uptake of recommended feeding and caring practices; learn more about the types of foods used for complementary feeding and the age of children when these foods are introduced; identify feasible and effective channels for promoting recommended infant and young child feeding practices.	Qualitative study (focus group discussion and in-depth interviews) AM: Formative research, Thematic analysis	Targeted recruitment and purposive sampling, 24 total FGDs with 6 FGDs in 4 groups (mothers with children < 6 months; mothers with children 6 to 23 months of age; fathers with children ≤ 23 months; grandmothers with children ≤ 23 months	5. no explanation regarding the data collection setting 6. no discussion about the researcher’s role, potential bias and influence
18	United States Agency for International Development 2011 Nigeria	Formative assessment of infant and young child feeding practices-Federal Capital Territory, Nigeria	United States Agency for International Development	“(1) To conduct a cross-sectional investigation of breastfeeding habits of caregivers among target groups. (2) To investigate complementary feeding practices among target groups. (3) To document responsive feeding practices, food hygiene, and feeding the sick child practices in the study areas. (4) To identify prospects of health communication interventions through the use of relevant media for reaching the respective target groups. (5) To identify existing alternative platforms for communication interventions and information sharing and dialogue among target groups.”	Qualitative study (focus group discussion with care givers) AM: Formative research, Thematic content analysis	Purposive sampling (from health facility), Focus group discussions (91 mothers of children < 6 months old; 81 mothers of children 6 to 24 months old; 75 grandmothers of children under-24-month-olds; and 82 fathers of children under-24- month-olds)	6. no discussion about the researcher’s role, potential bias and influence 7. no information about approval from the ethics committee
19	United States Agency for International Development 2011 Malawi	Consulting with caregivers –formative research to determine the barriers and facilitators to optimal infant and young child feeding in three regions of Malawi	United States Agency for International Development	Identify specific dietary problems among children less than 2 years of age, including determining the energy density and nutrient density (particularly iron, zinc, and vitamin A) of common foods given to children in different age groups. Gain a further understanding of current feeding practices and feeding problems that Impede adequate dietary intake in children less than 2 years of age. Describe maternal/child behaviors related to responsive feeding. Identify the positive and negative social, cultural, and economic factors that influence current practices, including the influence of other family and community members.	Qualitative study (In-depth interviews with care givers, observations, and trials of improved practices) AM: Formative research, Thematic content analysis	Purposive sampling, Interviews with 60 mothers/caregivers of children 6–23 months and 18 key informants	6. no discussion about the researcher’s role, potential bias and influence
20	Academy for Educational Development (AED)/Alive and Thrive Ethiopia Country Office 2011 Ethiopia	Initial Insight Mining and Pretest Research for Alive and Thrive Ethiopia	Academy for Educational Development (AED)/ Alive and Thrive Ethiopia Country Office	To assess IYCF practices as well as an analysis of the specific information gaps that needed to be explored through the insight mining and pretest research.	Qualitative study (focus group discussion with mothers/caregivers) AM: Not stated	Multi-stage purposive sampling, 32 focus group discussions among mothers, fathers and mothers in law	5. no explanation of data collection setting and the format of data 6. no discussion about the researcher’s role, potential bias and influence 7. no explicit information about approval from the ethics committee
21	United States Agency for International Development 2011 Kenya	Engaging grandmothers and men in infant and young child feeing and maternal nutrition—Report of a formative assessment in Eastern and Western Kenya	United States Agency for International Development	Assess complementary feeding practices of children younger than 2 years and community beliefs that influence these. Determine maternal dietary practices during pregnancy and lactation and household factors that influence these. Establish the roles and responsibilities of men and grandmothers in the family and in maternal dietary and infant and young child feeding practices. Determine cultural influencers of maternal, infant, and young child feeding. Document available services and support for nutrition in the intervention areas.	Qualitative study (focus group discussion with mothers/caregivers) AM: Formative research, Thematic content analysis	Purposive sampling, 16 focus group discussions groups: (fathers of children < 2 years; 9 mothers of children < 2 years; grandmothers of children <2 years; mothers of children < 2 years; and on maternal nutrition for mothers of children < 2 years)	6. no discussion about the researcher’s role, potential bias and influence
22	United States Agency for International Development 2011 Ghana	Ghana promotion of complementary feeding practices project–baseline survey report	United States Agency for International Development	To provide an in-depth analysis of infant and young child feeding knowledge, practices, and behaviors as a baseline to guide the implementation of the Ghana Promotion of Complementary Feeding Practices Project, and to provide a basis for monitoring and evaluating the effectiveness of the behavior change communication campaign	Mixed method study (in-depth interviews and focus group discussion) AM: Thematic analysis; triangulation of different data sources	Purposive and convenience selection, 20 focus group discussions with fathers of children 6–24 months and 30 focus group discussions with mothers/caregivers of children 6–24 months.	5. no explanation regarding the form of data) 6. no discussion about the researcher’s role, potential bias and influence 7. no information about approval from the ethics committee
23	Alive and Thrive Bangladesh 2012 Bangladesh	Perceptions, practices, and promotion of infant and young child feeding–results and program implications of assessments in Bangladesh	Alive and Thrive Bangladesh	“To determine the perceptions of mothers, influential family and community members and understanding of community-level health care providers of IYCF.” to assess constraints and motivators as well as sources of information on IYCF	Mixed method Study (in-depth interviews and focus group discussion) AM: Not stated	Purposive convenience sampling Semi-structured interviews with 42 mothers of children 0–23.9 months and 28 focus group discussions with fathers and grandmothers	5. no explanation regarding the form of data 6. no discussion about the researcher’s role, potential bias and influence 7. no information about approval from the ethics committee 8. no information about the process of analysis

**Table 2:** CERQual qualitative evidence profile of identified barriers and facilitators.

Finding No.	Review Finding	Studies Contributing to the Review Finding	Assessment of Methodological Limitations	Assessment of Relevance	Assessment of Coherence	Assessment of Adequacy	Overall CERQual Assessment of Confidence	Explanation of Judgement
**Barriers**								
**Barriers to Recommended Breastfeeding**								
Key Theme: *Beliefs and Perceptions*								
1	Breastfeeding alters a woman’s breasts in an undesirable way	1; 4; 13	Minor methodological limitations	Minor concerns about relevance	Minor concerns about coherence	No concern about adequacy of data	High confidence	This finding was graded as high confidence because of few concerns about coherence and relevance and moderate methodological limitations.
2	Breastfeeding for more than one year makes children susceptible to infection	11	Minor methodological limitations	NA	NA	Minor concern about adequacy	NA	NA
3	Breastmilk is unhealthy for baby if produced while a mother is angry	11	Minor methodological limitations	NA	NA	Minor concern about adequacy	NA	NA
4	Breastmilk is unhealthy for baby if produced while a mother has an illness	11	Minor methodological limitations	NA	NA	Minor concern about adequacy	NA	NA
5	Breastmilk is not enough for a small baby	8; 16	Substantial methodological limitations	Moderate concerns about relevance	Minor concerns about coherence	Moderate concerns about adequacy	Low confidence	This finding was graded as low confidence because of moderate concerns about relevance and adequacy of data and substantial methodological limitations.
6	When baby belches on the breast during breastfeeding the mother can develop swollen breasts	4	Moderate methodological limitations	NA	NA	No concern about adequacy of data	NA	NA
7	Colostrum is not healthy for baby, so mothers discard colostrum	3; 7; 10; 18	Moderate methodological limitations	Minor concerns about relevance	Minor concerns about coherence	Minor concerns about adequacy	Moderate confidence	This finding was graded as moderate confidence because of minor concerns regarding relevance, coherence, and adequacy and moderate methodological limitations.
8	Baby remains hungry after breastfeeding (e.g., a baby is fussy even after breastfeeding)	5; 6; 8; 9; 14; 15; 16; 17; 19; 21	Moderate methodological limitations	Minor concerns about relevance	Minor concerns about coherence	Moderate concerns about adequacy	Moderate confidence	This finding was graded as moderate confidence because of moderate concerns regarding methodological limitations and adequacy of data and minor concerns about relevance and coherence.
9	Breast milk substitutes can make a baby healthier	8	Moderate methodological limitations	NA	NA	No concern about adequacy	NA	NA
10	Mothers do not produce sufficient breastmilk	3; 4; 5; 6; 7; 8; 9; 13; 14; 16; 17; 19; 21; 23	Moderate methodological limitations	Minor concerns about relevance	Minor concerns about coherence	Minor concerns about adequacy	High confidence	This finding was graded as high confidence because of minor concerns about relevance, coherence and adequacy and moderate methodological limitations.
11	Babies are thirsty and need water	2; 7; 9; 13; 14; 16; 17; 19; 21; 23	Moderate methodological limitations	Minor concerns about relevance	Minor concerns about coherence	Moderate concerns about adequacy	Moderate confidence	This finding was graded as moderate confidence because of moderate concerns regarding methodological limitations and adequacy of data and minor concerns about relevance and coherence.
12	Other liquids given shortly after birth to clean the infant, believing the insides need to be purged	11	Minor methodological limitations	NA	NA	Substantial concerns about adequacy	NA	NA
13	Delay in breastmilk “coming in” following the birth (delayed onset of Lactogenesis II)	3	Moderate methodological limitations	NA	NA	No concern about adequacy of data	NA	NA
14	Giving traditional herbs to newborns or young babies is a deeply rooted practice	20	Moderate methodological limitations	NA	NA	Minor concerns about adequacy	NA	NA
15	Mothers do not have confidence that they are producing sufficient breast milk to meet baby’s needs	5	Moderate methodological limitations	NA	NA	No concern about adequacy of data	NA	NA
16	Baby still cries even after mother breastfeeds	21	Moderate methodological limitations	NA	NA	No concern about adequacy of data	NA	NA
17	Herbal infusions in water prevent a baby from getting ill	18	Moderate methodological limitations	NA	NA	No concern about adequacy of data	NA	NA
18	Exclusive breastfeeding is not part of local tradition	2; 7; 14; 16; 17; 18; 19; 21; 22	Moderate methodological limitations	Minor concerns about relevance	Moderate concerns about coherence	Moderate concerns about adequacy of data	Moderate confidence	This finding was graded as moderate confidence because of minor concerns about relevance and moderate concerns regarding methodological limitations and coherence and adequacy of data.
19	Mothers attribute their inability to produce enough breastmilk to their own diet	11; 13; 14; 17; 18; 21; 23	Moderate methodological limitations	Minor concerns about relevance	Minor concerns about coherence	Minor concerns about adequacy of data	High confidence	This finding was graded as high confidence because of minor concerns about relevance, coherence and adequacy and moderate methodological limitations.
20	Women should cease breastfeeding if they become pregnant before nursling is 2 years old	15; 17; 18; 21	Moderate methodological limitations	Minor concerns about relevance	Minor concerns about coherence	Moderate concerns about adequacy of data	Moderate confidence	This finding was graded as moderate confidence because of minor concerns about relevance, coherence and moderate concerns about methodological limitations and adequacy of data.
Key Theme: *Lack of Support from Families, Health Workers (HWs) and others*								
21	Family members do not support ideal breastfeeding practices	4; 19; 20; 21	Moderate methodological limitations	Minor concerns about relevance	Minor concerns about coherence	Substantial concerns about adequacy	Moderate confidence	This finding was graded as moderate confidence because of minor concerns about coherence and relevance; moderate methodological limitations; and substantial concerns about adequacy.
22	Mothers do not have enough time to breastfeed their infants due to work	4; 6; 7; 8; 9; 13; 14; 15; 16; 17; 18; 20	Moderate methodological limitations	Minor concerns about relevance	Minor concerns about coherence)	Moderate concerns about adequacy	Moderate confidence	This finding was graded as moderate confidence because of minor concerns about relevance and moderate concerns regarding methodological limitations and coherence and adequacy of data.
23	HWs do not reassure women about their ability to breastfeed	3	Moderate methodological limitations	NA	NA	No concern about adequacy of data	NA	NA
24	HWs do not have adequate knowledge and skills to support mothers	2; 9; 23	Moderate methodological limitations	Moderate concerns about relevance	Minor concerns about coherence	Minor concerns about adequacy	Moderate confidence	This finding was graded as moderate confidence because of minor concerns about coherence and adequacy; moderate concern about relevance and methodological limitations.
25	Mothers do not receive advice from HWs	3; 9; 10; 13	Minor methodological limitations	Minor concerns about relevance	Minor concerns about coherence	Minor concerns about adequacy	High confidence	This finding was graded as high confidence because of few concerns about methodological limitations, relevance, coherence and adequacy.
26	Media has a negative impact on breastfeeding (promoting breastmilk substitutes as healthy for babies)	7; 8; 13	Moderate methodological limitations	Minor concerns about relevance	Minor concerns about coherence	No concern about adequacy of data	High confidence	This finding was graded as high confidence because of few concerns about coherence and relevance and moderate methodological limitations.
27	Traditional beliefs and influence of older female family members (e.g., grandmothers) influence breastfeeding	7; 16; 18; 21	Moderate methodological limitations	Minor concerns about relevance	Minor concerns about coherence	Moderate concerns about adequacy	Moderate confidence	This finding was graded as moderate confidence because of minor concerns about coherence and relevance; moderate concern about methodological limitations and adequacy.
28	Comments/advice from families, peers or close neighbors influence breastfeeding	8; 9; 13	Moderate methodological limitations	Minor concerns about relevance	Minor concerns about coherence	No concern about adequacy of data	High confidence	This finding was graded as high confidence because of adequate data, minor concerns about coherence and relevance and moderate concerns about methodological limitations.
Key Theme: *Infant-Specific Factors*								
29	Babies stop breastfeeding for unknown reasons (lack of interest in feeding, self-weaning)	9; 13	Moderate methodological limitations	Moderate concerns about relevance	Minor concerns about coherence	No concern about adequacy of data	Moderate confidence	This finding was graded as moderate confidence because of minor concerns about coherence, moderate concern about relevance and methodological limitations.
30	Delayed breastfeeding initiation of infants with respiratory problems	10	Minor methodological limitations	NA	NA	No concern about adequacy of data	NA	NA
31	Baby has an illness	4; 10; 14; 17; 19	Moderate methodological limitations.	Minor concerns about relevance	Minor concerns about coherence	Substantial concerns about adequacy	Moderate confidence	This finding was graded as moderate confidence because of substantial concerns about adequacy; moderate methodological limitations; and minor concerns regarding coherence and relevance.
Key Theme: *Mothers’ Understanding*								
32	Mothers do not understand purpose of colostrum and breastfeeding initiation	3; 7; 8; 14; 17; 18; 20	Moderate methodological limitations	Minor concerns about relevance	Minor concerns about coherence	Substantial concerns about adequacy	Moderate confidence	This finding was graded as moderate confidence because of substantial concerns about adequacy; moderate methodological limitations; and minor concerns regarding coherence and relevance.
33	Mothers do not have technical knowledge about breastfeeding practices	8; 9; 10; 13; 14; 15; 17; 18; 20; 21; 22	Moderate methodological limitations	Minor concerns about relevance	Moderate concerns about coherence	Moderate concerns about adequacy	Moderate confidence	This finding was graded as moderate confidence because of moderate concerns regarding methodology, coherence, adequacy and minor concerns about relevance.
34	Mothers unable to interpret child’s feeding cues or behavior	8; 17; 19	Moderate methodological limitations	Moderate concerns about relevance	Minor concerns about coherence	Minor concerns about adequacy	Moderate confidence	This finding was graded as moderate confidence because of moderate concerns about methodology, relevance, coherence and minor concerns about adequacy.
35	Mothers do not have formal schooling or education	2	Moderate methodological limitations	NA	NA	Minor concern about adequacy of data	NA	NA
Key Theme: *Mother-Specific Factors*								
36	Mothers have breast or nipple problems	4; 5; 7; 9; 13; 14	Moderate methodological limitations	Minor concerns about relevance	Minor concerns about coherence	Minor concern about adequacy of data	High confidence	This finding was graded as high confidence because of minor concerns regarding relevance, coherence, adequacy and moderate methodological limitations.
37	Mothers have an illness and/or have died	4; 9; 12; 13; 14; 15; 17; 18; 21	Moderate methodological limitations	Minor concerns about relevance	Minor concerns about coherence	Substantial concerns about adequacy	Moderate confidence	This finding was graded as moderate confidence because of substantial concerns about adequacy of data, moderate methodological limitations and minor concerns regarding relevance and coherence.
38	Breastfeeding initiation is delayed due to pregnancy/labor-induced health conditions or activities needing to be performed after childbirth	3; 10; 19	Minor methodological limitations	Moderate concerns about relevance	Minor concerns about coherence	Substantial concerns about adequacy	Moderate confidence	This finding was graded as moderate confidence because of substantial concerns about adequacy, moderate concerns about relevance and minor concerns about coherence and methodological limitations.
**Barriers to Complementary Feeding**								
Key Theme: *Food Security and Social and Cultural Factors*								
39	Household food insecurity	11; 12; 14; 15; 16; 17; 20; 21; 23	Moderate methodological limitations	Minor concerns about relevance	Minor concerns about coherence	Minor concerns about adequacy	High confidence	This finding was graded as high confidence because of minor concerns about relevance, coherence and adequacy; and moderate methodological limitations.
40	Replacement of higher quality foods with lower quality foods for financial reasons or drought	12; 15	Moderate methodological limitations	Moderate concerns about relevance	Minor concerns about coherence	No concern about adequacy of data	Moderate confidence	This finding was graded as moderate confidence because of minor concerns about coherence, moderate concern about relevance and methodological limitations.
41	Lack of diversity in available foods	12; 14; 15; 16; 17; 19; 20	Moderate methodological limitations	Minor concerns about relevance	Minor concerns about coherence	Minor concerns about adequacy	High confidence	This finding was graded as high confidence because of minor concerns about relevance, coherence and adequacy; and moderate methodological limitations.
42	Lack of water for cooking	12; 15	Moderate methodological limitations	Moderate concerns about relevance	Minor concerns about coherence	No concern about adequacy of data	Moderate confidence	This finding was graded as moderate confidence because of minor concerns about coherence, moderate concern about relevance and methodological limitations.
43	Some foods are only available seasonally	12	Minor methodological limitations	NA	NA	Minor concern about adequacy of data	NA	NA
44	Mothers prepare foods early in the day and then store for later feeding	12	Minor methodological limitations	NA	NA	No concern about adequacy of data	NA	NA
45	Mothers do not have financial decision-making power	14; 20	Moderate methodological limitations	Moderate concerns about relevance	Minor concerns about coherence	No concern about adequacy of data	Moderate confidence	This finding was graded as moderate confidence because of minor concerns about coherence, moderate concern about relevance and methodological limitations.
Key Theme: *Infant-Specific Factors*								
46	Infants dislike animal source foods	6; 23	Moderate methodological limitations	Moderate concerns about relevance	Minor concerns about coherence	Substantial concern about adequacy	Low confidence	This finding was graded as low confidence because of moderate concerns about methodology and relevance; and substantial concerns about adequacy of data.
47	Infants do not have appetite to eat animal source foods	6; 18	Moderate methodological limitations	Moderate concerns about relevance	Minor concerns about coherence	Moderate concern about adequacy	Moderate confidence	This finding was graded as moderate confidence because of moderate concerns regarding methodological limitations, adequacy of data, relevance and minor concerns about coherence.
48	Infants refuse to eat and or spit out foods	6; 15; 23	Moderate methodological limitations	Moderate concerns about relevance	Moderate concern about coherence	Substantial concerns about adequacy	Low confidence	This finding was graded as low confidence because of moderate concerns about relevance and coherence and substantial concerns about adequacy of data and methodology.
49	Infants vomit when animal source foods are offered	6; 15	Substantial methodological limitations	Moderate concerns about relevance	Moderate concern about coherence	Substantial concerns about adequacy	Low confidence	This finding was graded as low confidence because of moderate concerns about relevance and coherence and substantial concerns about adequacy of data and methodology.
50	Infants have an illness	15; 18	Moderate methodological limitations	Moderate concerns about relevance	Minor concerns about coherence	No concern about adequacy of data	Moderate confidence	This finding was graded as moderate confidence because of moderate concerns about methods and methodology and minor concerns about coherence.
Key Theme: *Family-Specific Factors*								
51	Mothers and other family members do not have appropriate knowledge about complementary feeding	6; 11; 14; 15; 16; 19; 20; 21; 23	Moderate methodological limitations	Minor concerns about relevance	Minor concerns about coherence	Minor concerns about adequacy	High confidence	This finding was graded as high confidence because of minor concerns about relevance, coherence and adequacy; and moderate methodological limitations.
52	Mothers concern about over-feeding their child (which might cause them to be hungrier in future)	16	Moderate methodological limitations	NA	NA	No concern about adequacy of data	NA	NA
53	Mothers and family members fear that child may choke on certain solid foods	15; 16; 19	Moderate methodological limitations	Moderate concerns about relevance	Minor concerns about coherence	Moderate concern about adequacy of data	Moderate confidence	This finding was graded as moderate confidence because of moderate concerns about methodology, adequacy of data, relevance and minor concern regarding coherence.
54	Religious/cultural traditions around preparation of non-fasting foods for children by adults who are fasting	15	Moderate methodological limitations	NA	NA	Moderate concern about adequacy of data	NA	NA
55	Thicker weaning porridge believed to be difficult for young children to swallow/digest	15; 16; 19; 20; 23	Moderate methodological limitations	Moderate concerns about relevance	Minor concerns about coherence	Moderate concern about adequacy of data	Moderate confidence	This finding was graded as moderate confidence because of moderate concerns about methodology, adequacy of data, relevance and minor concern regarding coherence.
56	Lack of time to cook special foods for the child	15; 16	Moderate methodological limitations	Moderate concerns about relevance	Minor concerns about coherence	Minor concern about adequacy of data	Moderate confidence	This finding was graded as moderate confidence because of moderate concerns about methodology and relevance and minor concerns regarding coherence and adequacy of data.
**Cross-Cutting Barriers**								
57	Inability of health workers to provide optimal support to mothers	15	Moderate methodological limitations	NA	NA	No concerns about adequacy of data	NA	NA
58	Conflicting information	21	Moderate methodological limitations	NA	NA	Moderate concerns	NA	NA
**Facilitators**								
**Facilitators to Recommended Breastfeeding**								
Key Theme: *Perceptions*								
59	Breastfeeding is inexpensive and hygienic	1; 2; 4; 7; 9; 13	Moderate methodological limitations	Minor concerns about relevance	Minor concerns about coherence	Minor concerns about adequacy	High confidence	This finding was graded as high confidence because of minor concerns about relevance, coherence and adequacy; and moderate methodological limitations.
60	Breastfeeding relieves uncomfortable fullness of breasts	4	Moderate methodological limitations	NA	NA	Moderate concerns about adequacy	Low confidence	This finding was graded as low confidence because of moderate concerns about adequacy and minimal studies contributing evidence.
61	Breastfeeding strengthens the bond between the mother and child	5; 9	Moderate methodological limitations	Moderate concerns about relevance	Minor concerns about coherence	Moderate concerns about adequacy	Moderate confidence	This finding was graded as moderate confidence because of moderate concerns regarding methodology, relevance and adequacy and minor concerns about coherence.
Key Theme: *Healthcare Services*								
62	Advice and encouragement provided in some healthcare setting(s) (e.g., prenatal visits, delivery, home visits)	3; 10; 13; 16; 17; 18	Moderate methodological	Minor concerns about relevance	Minor concerns about coherence	Substantial concerns about adequacy	Moderate confidence	This finding was graded as moderate confidence because of substantial concerns about adequacy, moderate methodological limitations, minor concerns about relevance and coherence.
63	Some advice and counseling from health workers on importance of breastfeeding	2; 3; 4; 9; 13; 14; 15; 16; 17; 18; 19	Moderate methodological limitations	Minor concerns about relevance	Minor concerns about coherence	Substantial concerns about adequacy	Moderate confidence	This finding was graded as moderate confidence because of substantial concerns about adequacy, moderate methodological limitations, minor concerns about relevance and coherence.
Key Theme: *Infant-Specific Factors*								
64	Child crying provides guidance when to feed	2; 10	Minor methodological limitations	Moderate concerns about relevance	Minor concerns about coherence	Minor concerns about adequacy	High confidence	This finding was graded as high confidence due to only minor concerns about adequacy, relevance and methodological limitations and moderate concerns about relevance.
Key Theme: *Mother-Specific Factors*								
65	Mothers understand the importance of breastfeeding	1; 2; 3; 4; 5; 7; 9; 10; 11; 15; 16; 17; 18; 19; 21; 22	Moderate methodological limitations	Minor concerns about relevance	Minor concerns about coherence	Minor concerns about adequacy	High confidence	This finding was graded as high confidence because of minor concerns about relevance, coherence and adequacy; and moderate methodological limitations.
66	Mothers understand the importance of colostrum	2; 7; 14; 19	Moderate methodological limitations	Minor concerns about relevance	Minor concerns about coherence	Minor concerns about adequacy	High confidence	This finding was graded as high confidence because of minor concerns about relevance, coherence and adequacy; and moderate methodological limitations.
Key Theme: *Social and Cultural Factors*								
67	Cultures and traditions facilitate early initiation of breastfeeding	3	Moderate methodological limitations	NA	NA	No concerns about adequacy	Low confidence	This finding was graded as low confidence because of minimal studies contributing evidence.
68	Family support promotes breastfeeding	1; 2; 7; 9	Moderate methodological limitations	Minor concerns about relevance	Minor concerns about coherence	Substantial concerns about adequacy	Moderate confidence	This finding was graded as moderate confidence because of moderate methodological limitations, minor concerns regarding relevance and coherence and substantial concerns about adequacy.
69	Praise and affirmation from a partner and/or family facilitate breastfeeding	2	Moderate methodological limitations	NA	NA	No concerns about adequacy	NA	NA
**Facilitators to Complementary Feeding**								
Key Theme: *Food Security*								
70	Some healthy foods are available	16; 18; 22	Moderate methodological limitations	Moderate concerns about relevance	Minor concerns about coherence	Minor concerns about adequacy	Moderate confidence	This finding was graded as moderate confidence because of moderate concerns about methodology and relevance; and minor concerns about coherence and adequacy.
Key Theme: *Social Support*								
71	Family support promotes complementary feeding	6; 15; 16; 21	Moderate methodological limitations	Moderate concerns about relevance	Minor concerns about coherence	Minor concerns about adequacy	Moderate confidence	This finding was graded as moderate confidence because of minor concerns about coherence and adequacy; and moderate concerns regarding relevance and methodology.
Key Theme: *Mother-Specific Factors*								
72	Mothers understand the importance of complementary feeding	15; 16; 18	Moderate methodological limitations	Moderate concerns about relevance	Minor concerns about coherence	Minor concerns about adequacy	Moderate confidence	This finding was graded as moderate confidence because of minor concerns about coherence and adequacy; and moderate concerns.
Key Theme: *Healthcare Services*								
73	Mothers receive IYCF advice and counseling at healthcare facilities	15; 18; 19; 21	Moderate methodological	Minor concerns about relevance	Minor concerns about coherence	Substantial concerns about adequacy	Moderate confidence	This finding was graded as moderate confidence because of minor concerns about coherence and adequacy; and moderate methodological limitations and substantial concerns about adequacy.

NA: not applicable.

**Table 3:** CERQual summary of qualitative review findings.

Objective:	To systematically review qualitative literature related to family experiences (particularly parental ones) of infant and young child feeding in low-income and lower-middle income countries, synthesizing information on the barriers and facilitators that may relate to interventions to impact nutrition, survival, growth and development.			
Perspective:	Experiences, beliefs, attitudes and perceptions of parents/caregivers who routinely engage in infant and young child feeding.			
Included studies:	Included studies involved participants from low-income or lower-middle income countries who were parents or close family members of an infant or young child (0–2 years of age) at the time of the study; used widely accepted qualitative data collection methods; and provided a clear description of recognized qualitative data analysis methods.			
Key theme	Synthesized Review Finding	Emergent findings incorporated into synthesized finding (finding numbers from Table 2)	Studies contributing evidence for review finding (study numbers from Table 1)	Overall CERQual assessment of confidence
**Barriers**				
Barriers to Recommended Breastfeeding Practices				
*Beliefs and perceptions*	Breastfeeding alters a woman’s breasts in an undesirable way	1	1; 4; 13	High confidence
	Traditional beliefs indicate that colostrum is not good for the baby, or that breastfeeding should be delayed until more white milk comes in • Colostrum is not healthy for baby, so mothers discard it • Delay in breastmilk “coming in” following the birth (delayed onset of Lactogenesis II)	7; 13	3; 7; 10; 18	Moderate confidence
	The breastmilk produced by mother is not sufficient for the infant • not enough water or sustenance in the breastmilk • babies are thirsty and need water • baby still cries even after mother breastfeeds (so caregivers believe baby must still be hungry or thirsty) • mothers do not have confidence that they are producing sufficient breast milk to meet baby’s needs	5; 8; 10; 11; 15; 16	2; 3; 4; 5; 6; 7; 8; 9; 13; 14; 15; 16; 17; 19; 21; 23	Moderate/High confidence
	Traditional foods/drinks are believed to be good for baby’s health and encouraged by older influential family members, undermining exclusive breastfeeding	12; 14; 17; 18; 27; 28	2; 7; 11; 14; 16; 17; 18; 19; 20; 21; 22	Moderate/High confidence
	Women should cease breastfeeding if they become pregnant before nursling is 2 years old	20	15; 17; 18; 21	Moderate confidence
	Breast milk substitutes can make a baby healthier/Media has a negative impact on breastfeeding (promoting breastmilk substitutes as healthy for babies)	9; 26	7; 8; 13	High confidence
*Lack of support*	Family, peers or close neighbors do not support, or undermine optimal breastfeeding practices: • Family members do not support ideal breastfeeding practices • Traditional beliefs and influence of older female family members (e.g., grandmothers) influence breastfeeding • Comments/advice from families, peers or close neighbors influence breastfeeding	21; 27; 28	4; 8; 9; 13; 19; 16; 18; 20; 21	Moderate/High confidence
	Lack of support from HWs • HWs do not reassure women about their ability to breastfeed • HWs do not have adequate knowledge and skills to support mothers • Mothers do not receive advice from HWs	23; 24; 25	2; 3; 9; 10; 13; 23	Moderate/High confidence
	Mothers do not have enough time to breastfeed their infants due to work • maternity leave is short • minimal acceptance of expressing breastmilk • unwillingness to breastfeed in public—not wanting to expose breasts	22	4; 6; 7; 8; 9; 13; 14; 15; 16; 17	Moderate confidence
*Infant-specific factors*	Baby stops or is unable to breastfeed: • baby has an illness • babies stop breastfeeding for unknown reasons (lack of interest in feeding, self-weaning)	29; 30; 31	9; 4; 10; 13; 14; 17; 19	Moderate confidence
*Mother-specific factors*	Lack of knowledge and education about breastfeeding which influences her self confidence • Mothers do not understand purpose of colostrum and breastfeeding initiation • Mothers do not have knowledge about breastfeeding practices generally • Mothers do not have formal schooling or education	32; 33; 35	2; 3; 7; 8; 9; 10; 13; 14; 15; 17; 18; 20; 21; 22	Moderate confidence
	Mothers unable to interpret child’s feeding cues or behavior	34	8; 17; 19	Moderate confidence
	Mother’s health problems influence breastfeeding: • illness • death • breast or nipple problems (swollen and painful breasts, sore nipples, breast abscesses) • childbirth complications (causing delay in initiation of breastfeeding)	36; 37; 38	3; 4; 5; 7; 9; 10; 13; 14; 15; 17; 18; 19; 21	Moderate/High confidence
	Mothers attribute their inability to produce enough breastmilk to their own diet	19	4; 11; 13; 14; 17; 18; 21; 23	High confidence
Barriers to Recommended Complementary Feeding Practices				
*Food security and social and cultural factors*	Household food insecurity; • replacement of higher quality foods with lower quality foods for financial reasons or drought	39; 40	11; 12; 14; 15; 16; 17; 20; 21; 23	High confidence
	Lack of diversity in available foods; food shortages	41	12; 14; 15; 16; 17; 19; 20	High confidence
	Lack of water for cooking	42	12; 15	Moderate confidence
	Mothers lack financial decision making power, inhibiting ability to buy food	45	14; 20	Moderate confidence
*Infant-specific factors*	Animal source foods are not suitable for infants: • infants do not like animal source foods • infants vomit when given animal source foods • infants lack appetite for animal source foods • animal source foods are not appropriate for infants	46; 47; 49	6; 15; 18; 23	Low/Moderate confidence
	Infants refuse to eat and or spit out foods	48	6; 15; 23	Low confidence
	Infants have an illness	50	15; 18	Moderate confidence
*Family factors*	Mothers and other family members do not have appropriate knowledge about complementary feeding	51	6; 11; 14; 15; 16; 19; 20; 21; 23	High confidence
	Mother and other family members do not feel that baby can eat solid foods • fear of choking (e.g., on meat) • thicker weaning porridge believed to be difficult for young children to swallow/digest • lack of knowledge and self confidence in how to make foods appropriate for young child	53; 55	15; 16; 19; 20; 23	Moderate confidence
	Lack of time to cook special foods for the child	56	15; 16	Moderate confidence
Facilitators				
Facilitators to Recommended Breastfeeding Practices				
*Perceptions*	Breastfeeding is good for baby: makes baby grow strong, healthy and intelligent, prevents and shortens duration of illness, etc.	65	1; 2; 3; 4; 5; 7; 9; 10; 11; 15; 16; 17; 18; 19; 21; 22	High confidence
	Breastfeeding is inexpensive and hygienic	59	1; 2; 4; 7; 9; 13	High confidence
*Healthcare services*	Advice and encouragement provided in some healthcare setting(s) (e.g., prenatal visits, delivery, home visits); Some advice and counseling from health workers on importance of breastfeeding	62; 63	2; 3; 4; 9; 10; 13; 14; 15; 16; 17; 18; 19	Moderate confidence
*Infant-specific factors*	Child crying provides guidance when to feed	64	2; 10	High confidence
*Mother-specific factors*	Breastfeeding relieves the fullness of mothers’ breasts	60	4	Low confidence
	Breastfeeding strengthens mothers bond with infant	61	5; 9	Moderate confidence
	Mother understands the importance of colostrum	66	2; 7; 14; 19	High confidence
*Social and cultural factors*	Cultures and traditions facilitate early initiation of breastfeeding	67	3	Low confidence
	Family supports recommended breastfeeding practices	68; 69	1; 2; 7; 9	Moderate confidence
Facilitators to Recommended Complementary Feeding Practices				
*Food security*	Some healthy foods are available	70	16; 18; 22	Moderate confidence
*Social support*	Family supports recommended complementary feeding practices	71	6; 15; 16; 21	Moderate confidence
*Mother-specific factors*	Mother knows the importance of proper complementary feeding practices	72	15; 16; 18	Moderate confidence
*Healthcare services*	Mothers receive IYCF advice and counseling at healthcare facilities	73	15; 18; 19; 21	Moderate confidence

## Supplementary Materials

The following are available online at www.mdpi.com/2072-6643/9/10/1140/s1, Table S1: ENTREQ checklist (Enhancing transparency in reporting the synthesis of qualitative research).

## Appendix A

Details of search strategy.

**Table:**

Database	Date of Search	Search Terms	Total Articles Returned
Pubmed	9 July 2016	“infant nutritional physiological phenomena” (MeSH Terms) AND “qualitative research” (MeSH Terms) AND (“13 July 2006” (PDat): “9 July 2016” (PDat) AND “humans” (MeSH Terms) AND English (lang))	180 articles
Ebsco	9 July 2016	((MH “Infant Nutritional Physiology+”) OR (MH “Infant Feeding+”)) AND Qualitative studies Limiters-Published Date: 1 January 2006–31 December 2016; English Language; Abstract Available; Human; Language: English Search modes-Find all my search terms	287 articles
Embase	9 July 2016	‘infant feeding’/exp OR ‘infant feeding’ OR ‘complementary feeding’/exp OR ‘complementary feeding’ AND (‘qualitative research’/exp OR ‘qualitative research’) AND (embase)/lim AND (2006–2016)/py AND (humans)/lim	151 articles

## Appendix B

**Table:**

Study No.	Author and Title	Participant Characteristics	Key Outcomes Reported	Emergent Findings (Finding Numbers from Table 2)
*Studies Identified from Search Databases*				
1	Lynn M. Babington Understanding Beliefs, Knowledge, and Practices of Mothers in the Dominican Republic Related to Feeding Infants and Young Children	Mothers’ ages ranged from 16 to 45 (mean age = 25), “The highest educational level attained was as follows: one had attended university (did not graduate), two had graduated from secondary school, one had completed eighth grade, three had completed sixth grade, two had completed fourth grade, and one had never attended school. When asked how well they spoke and/or understood English, two said “a little,” and the other eight answered “not at all.” The estimated yearly income for the entire family was reported by all 10 participants as less than 28,500 pesos ($868 U.S. dollars).”	*From abstract:* “The women who participated in the focus group all breast fed their babies with the belief that breast milk is better for infants than formula. Cereal, fruits, and vegetables are the main diet for children under 3 years old. Typically, “fast foods” and snack foods are not readily available and therefore they are rarely consumed. Obesity was viewed as a health risk for children. These mothers were practicing many healthy feeding behaviors, such as breastfeeding and limiting fats and sweets for young children, and they recognized their importance in promoting the health of children.” *Additional notes:* Participants universally practiced breastfeeding in part because formula is expensive and not readily available. They recognized the association between breastfeeding and the health of their babies. They all learned to breastfeed from their mothers/grandmother/sisters and said that everyone in the DR learns this way. Older mothers mentioned that breastfeeding has a negative effect on the physical appearance of their breasts but that this did not impact their decision to breastfeed because formula is not affordable. Mothers said they avoid giving fast foods and snack foods (which are not readily available), greasy/fatty foods, and sweats to young children. Since they do not give greasy/fatty foods to young children, they do not give much meat until around 3 years because it is greasy. Participants said that 3 years is the age when children eat what everyone else in the house eats in the DR.	(1) (59) (65) (68)
2	Amal Omer-Salim, Lars-Ake Persson, PiaOlsson Whom can I rely on? Mothers’ approaches to support for feeding: An interview study in suburban Dar es Salaam, Tanzania	Mothers were aged 16–30 years. Four women had no formal education, two had completed primary education and two had completed secondary education. Five of the women were homemakers and three were either formally employed or self-employed	*From abstract:* “The study revealed four categories of mothers’ perceptions of baby feeding: (1) baby feeding, housework and paid work have to adjust to each other; (2) breast feeding has many benefits; (3) water or breast milk can be given to quench baby’s thirst; and (4) crying provides guidance for baby feeding. Four different themes describing approaches to support emerged from the data: (1) adhering to diverse sources; (2) relying wholeheartedly on a mother figure; (3) working as a parental team; and (4) making arrangements for absence from the child.” *Additional notes:* Mothers believed breastfeeding makes babies healthy and prevents diseases. Also, breastfeeding was viewed as a cheap and hygienic option. Some believe colostrum is good for the baby and so initiated breastfeeding soon after birth, but some women did not believe this and discard it. Good nutrition for the mother thought to improve breastmilk output. Some women practice exclusive breastfeeding believing that breastmilk is all a baby needs, but some think the baby also needs water (but they know that the water must be clean to give to baby). It is a traditional practice to give boiled water shortly after birth. Some believe that giving water relieves baby from constipation. Initial crying was recognized as a prompt for breastfeeding, but too much crying led mother to give water or porridge and was seen as a sign of hunger (traditional beliefs say porridge will solve this and baby will stop crying). Advice from HWs was sometimes inadequate (too general, or infeasible for mother). Advice from mother/mother-in-law/senior mother figure sometimes refutes the advice given by HWs. Support and agreement from the husband encourages breastfeeding. Praise from partner or HW encouraged mother to continue to exclusively breastfeed. Work interrupted optimal baby feeding for many mothers, but some mothers reported expressing breastmilk into a clean cup while working to continue to breastfeed the baby.	(11) (18) (24) (35) (59) (63) (64) (65) (66) (68) (69)
3	C Tawiah-Agyemang, BR Kirkwood, K Edmond, A Bazzano and Z Hill Early initiation of breast-feeding in Ghana: barriers and facilitators	Of the 52 recently delivered mothers: age ranged from under 20 to 45. The majority of them lived in rural areas (*n* = 43, 83%). Nearly half the mothers did not complete any education (*n* = 24, 46%); 10 attained primary school; and 18 had attained education beyond primary school.	*From abstract:* “The major reasons for delaying initiation of breast-feeding were the perception of a lack of breast milk, performing postbirth activities such as bathing, perception that the mother and the baby need rest after birth and the baby not crying for milk. Facilitating factors for early initiation included delivery in a health facility, where the staff encouraged early breastfeeding, and the belief in some ethnic groups that putting the baby to the breast encourages the milk.” *Additional notes:* Barriers for delayed initiation: The perception of a lack of breastmilk: including physical signs that milk is absent/insufficient, beliefs about colostrum, and traditional beliefs of when the milk comes in (believed to be on the third day for some cultural groups in the study). Some believed colostrum was not good for the baby and so discarded it or delayed initiation until white milk came in. Delayed initiation of breastfeeding beyond 12 h often led to prelacteal feeding. Facilitators: Delivery at a health facility facilitated early initiation of breastfeeding due to advice of HWs.	(7) (10) (13) (23) (25) (32) (38) (62) (63) (65) (67)
4	Gloria E. Otoo, Anna A. Lartey, Rafael Pérez-Escamilla Perceived Incentives and Barriers to Exclusive Breastfeeding Among Peri-urban Ghanaian Women	The ages of the 35 participants ranged from 19 to 49 (mean age was 27.5). They had 7.2 ± 3.6 years of formal education. All but 3 of the women were employed, with about half (*n* = 17) being traders.	*From abstract:* “Almost all of the participants believed that exclusive breastfeeding is the superior infant feeding method and should be practiced for the first 6 months postpartum. However, there was widespread belief that infants can be given water if it is clean. Mothers reported that exclusive breastfeeding was easier when breast milk began to flow soon after delivery. The main obstacles to exclusive breastfeeding identified were maternal employment, breast and nipple problems, perceived milk insufficiency, and pressure from family.” *Additional notes:* Mothers obtained advice on exclusive breastfeeding (EBF) at the antenatal care clinic, during hospital stay, or at the postnatal care clinics. (Nurses advised them of the recommended practices, even if the reasons they gave were inaccurate). Knowledge that “breastmilk is best” was a motivation for women to practice EBF. Facilitators to EBF: Observation that EBF resulted in less frequent and shorter duration of illness in infants was a motivator for EBF. Mothers felt that EBF makes babies grow strong, intelligent, and healthy. Young babies stomachs are not mature enough for anything other than breastmilk (this finding lacks some coherence as some women believed breastmilk alone was not sufficient–e.g., when a baby still cries or remains restless even after breastfeeding it is a sign they need other foods). Mothers believed that her nutrition influences breastmilk production and quality; cultural beliefs influenced what foods they should eat for improved breastmilk production. Mothers’ knowledge that poor water and sanitation could cause a child to get diarrhea if child had other water or foods was a motivator for EBF. Some believed water should not be given to babies because of (runoff) from the fertilizers and chemicals used in the agricultural industry contaminating their water supply. Barriers to EBF: Swollen breasts, abscesses, and sore nipples are barriers to breastfeeding. Work interferes with breastfeeding (short maternity leave, followed by inability to find a place to breastfeed when they return to work). Maternal sickness and death prevents breastfeeding (though only two participants said women with HIV cannot breastfeed). Infant sickness impairs breastfeeding (child may lose interest or not latch on), along with weak jaws which impact sucking ability of infant, prompting mother to give other food so baby does not lose weight. Some mothers believe breastfeeding makes their breasts sag and so refuse to breastfeed. Women are hesitant to breastfeed in public, even in their cars because they do not want people to see their breasts. Pressure from mothers/grandmothers to adopt the mixed feeding methods their generation had used impacts EBF (especially if grandmother is around when infant is crying, they assume breastmilk is not enough), then women resort to adding water or not practicing EBF. Lack of support from the baby’s father could also hinder breastfeeding.	(1) (6) (10) (21) (22) (31) (36) (37) (59) (60) (63) (65)
5	Luzmil A Hernandez, Marth A Lucia Vasqu Practices and beliefs about exclusive breastfeeding by women living in Commune 5 in Cali, Colombia	The women participating ranged from 20 to 33 years of age. Regarding the level of education, one of the women had done university studies, four had completed high school, 5 had not finished high school, and the remaining participants had been in grammar School.	*From abstract:* “Findings are presented in two parts: practices and beliefs in favor of exclusive breastfeeding and practices and beliefs that do not support exclusive breastfeeding. The prominent practices and beliefs in favor of exclusive breastfeeding are related to the mother´s bond with the child, preparation for breastfeeding during pregnancy, and family support. Among the practices and beliefs not supporting maternal breastfeeding, we must highlight the mother’s lack of confidence in her breast milk production.” *Additional notes:* Breastfeeding was associated with providing love to their child and strengthening their bond. Women were convinced that breastfed children will be healthier and smarter in the future. But, exclusive breastfeeding for six months was considered difficult, and required preparation. They want their milk to be of adequate quality and quantity for their child. Some grandmothers or other elders had ideas of ways mothers could improves these milk traits (e.g., consuming fennel water). Mothers, mothers-in-law and grandmothers helped prepare mothers to breastfeed through their own experiences. Some still felt that breastmilk alone would not be enough for their child. Especially if the child continued crying. Pain in the breast was another reason for not exclusively breastfeeding. Some mothers who exclusively breastfed reported their child had become dependent on the breast and it was then difficult to wean. Along this line, mothers believed that weaning would be easier if the child had already had milk from other sources. There is a belief that children are born with weak stomachs and something must be done to strengthen it and protect them from disease. Home preparations are often given tot eh child within days after birth for this purpose; these included bean tincture, bacon, or a liquid to purge the stomach.	(8) (10) (15) (36) (61) (65)
6	Sabrina Rasheed, Rukhsana Haider, Nazmul Hassan, Helena Pachon, Sanjeeda Islam, Chowdhury S.B. Jalal, Tina G. Sanghvi Why does nutrition deteriorate rapidly among children under 2 years of age? Using qualitative methods to understand community perspectives on complementary feeding practices in Bangladesh	Not stated	*From abstract:* “Lay perceptions about complementary feeding differ substantially from international complementary feeding recommendations. A large proportion of children do not consume sufficient amounts of complementary foods to meet their energy and micronutrient needs. There was a gap in knowledge about appropriate complementary foods in terms of quality and quantity and strategies to convert family foods to make them suitable for children. Complementary feeding advice from family members, peers, and health workers, the importance given to feeding young children, and time spent by caregivers in feeding influenced the timing, frequency, types of food given, and ways in which complementary feeding occurred.” *Additional notes:* Children were fed complementary foods prior to 6 months of age primarily due to the perception that breastmilk alone was inadequate as the child got older. Liquids were given to young babies, including formula, which was considered good to maintain the health of the baby.	(8) (10) (22) (46) (47) (48) (49) (51) (71)
7	Pranee C. Lundberg, Trieu Thi Ngoc Thu Breast-feeding attitudes and practices among Vietnamese mothers in Ho Chi Minh City	Age range: 25 to 40 years Range of education levels: primary school to bachelors degree Majority lived in extended families and they had one to three children.	*From abstract:* “Five categories of breastfeeding attitudes and practices were identified: breast-feeding best but not exclusive, cultural and traditional beliefs, infant feeding as a learning process, factors influencing decision to breastfeed, and intention to feed the child.” *Additional notes:* Mothers recognized that breastmilk is best, but none were exclusively breastfeeding. Many gave water because they think the baby gets thirsty and that water will prevent tongue disease. Most discarded colostrum thinking it is harmful for baby, but a few knew that it was beneficial to baby’s health. Some were afraid breastmilk alone is not enough for baby so they also gave formula. Mothers believed that breastmilk quality was linked to health of mother (an ill mother would produce inferior milk), and her ability to consume the traditional postnatal diet. Mothers followed traditional beliefs (e.g., what to eat and not eat; messaging) to improve breastmilk production. Mothers, mothers-in-law, and grandmothers encouraged breastfeeding, told them how to breastfeed and helped with housework to allow mother to rest (after childbirth) and care for infant. The process of learning about breastfeeding from various sources gave them increased knowledge, which lead to improved self-confidence. Husband support gave confidence in breastfeeding. Returning to work shortly after birth (mostly before 6 months) made breastfeeding difficult. Media influenced mother’s opinions of formula and baby foods and made them think they are good for baby. Lactation problems (sore and cracked nipples, engorgement, inadequate lactation) interrupted breastfeeding with formula as a replacement. Size of baby influenced breastfeeding: mothers said they would feed baby more if he/she was small. Some mothers fed baby when he/she cried. After 4 months, mothers believed breastmilk alone was not enough so would add formula and/or complementary foods.	(7) (10) (11) (18) (22) (26) (27) (32) (36) (59) (65) (66) (68)
8	Yati Afiyanti, Dyah Juliastuti Exclusive breastfeeding practice in Indonesia	Mothers were aged 19 to 36 years	The initial breastfeeding practice immediately after delivery impeded the mothers’ decisions to exclusively breastfeed. For example, some women believed their baby had been given formula by the health provided after birth, so they hesitated to breastfeed exclusively. The belief of insufficient production of breast milk lead mothers to wean their babies early. After weaning their babies, the mothers still had a desire to breastfeed. Mothers kept trying to breastfeed even though they had weaned their baby and reported breastmilk production had decline. Early weaning caused several consequences (e.g., decline in breastmilk, increase in spending, health problems for the baby), which continued to affect the mother’s and baby’s lives. Some women though their babies grew faster after weaning and they enjoyed the extra time they had to spend on their daily activities. Media promotion of formula, suggestions from peers and family, and the mothers need to work were reported as external factors which impeded exclusive breastfeeding. Internal factors which did this included lack of knowledge about exclusive breastfeeding, techniques to ensure effective breastfeeding (e.g., ensuring baby was satiated), not recognizing baby feeding cues and behaviors, and believing formula can make babies healthier.	(5) (8) (9) (10) (22) (26) (28) (32) (33) (34)
9	Hope Mei Hong Lee, Jo Durham, Jenny Booth, Vanphanom Sychareun A qualitative study on the breastfeeding experiences of first-time mothers in Vientiane, Lao PDR	Mothers’ ages ranged from 19 to 39. Three had attained primary education; 5 had completed lower secondary education; 5 had attained; and 3 had finished tertiary education. The majority of them were unemployed and/or housewife (*n* = 9). Six were employed.	*From abstract:* “Participants demonstrated positive attitudes towards breastfeeding and recognized its importance. Despite this, breastfeeding practices were suboptimal. Few exclusively breastfed for the first six months of the baby’s life and most of the first-time mothers included in the sample had stopped or planned to stop breastfeeding by the time the infant was 18 months of age. Work was named as one of the main reasons for less than ideal breastfeeding practices. Traditional beliefs and advice from health staff and the first-time mothers’ own mothers, were important influences on breastfeeding practices. First-time mothers also cited experiencing tension when there were differences in advice they received from different people.” *Additional notes:* Health staff assisted women to introduce breastmilk early, and many reported the importance of the child receiving the colostrum. Breastfeeding was also reported to strengthen the bond between mother and newborn. Breastfeeding was thought to save money, giving it an advantage. Family elders have important role in decisions related to child feeding and one elder thought it was important to include them to avoid conflicts in advice given by health staff. Some women reported inconsistent advice from these two groups. Some women had trouble managing exclusive breastfeeding and work, with many citing it as the main reason for stopping exclusive breastfeeding early. Support from family and support in the workplace were also cited as reasons for success in exclusive breastfeeding. Insufficient milk supply, compressed (inverted) nipple and the baby just stopping sucking were all reported reasons for ending exclusive breastfeeding early. Fear of breast deformation and embarrassment of breastfeeding at social functions were also cited as reasons for stopping breastfeeding. Elders in the family often advocated for early introduction of complementary foods despite this being contradictory to the advice of health personnel.	(8) (10) (11) (22) (24) (25) (28) (29) (33) (36) (37) (59) (61) (63) (65) (68)
10	Christy Bomer-Norton Timing of breastfeeding initiation in rural Haiti: A focused ethnography	The range of mothers’ ages was between 19 years and 43 years. Years of education attainment ranged from 0 to 13 years.	*From abstract:* “Mothers described baby care by birth attendants as well as actual and perceived health issues of the mother and child as some of the factors influencing timing of breastfeeding initiation.” *Additional notes:* All 25 mothers believed that breastfeeding, colostrum and mature milk, was good for baby’s health and they value providing these health benefits to their baby; baby’s health was a priority for mothers. Mothers exhibited comfort with breastfeeding (comfortable feeding in front of others) and had the feeling that breastfeeding was the normal way of infant feeding.	(7) (25) (30) (31) (33) (38) (62) (64) (65)
11	Anne Laterra, Mohamed A. Ayoya, Jean-Max Beauliere, M’mbakwa Bienfait, Helena Pachon Infant and young child feeding in four departments in Haiti; mixed-method study on prevalence of recommended practices and related attitudes, beliefs, and other determinants	Note: this was a mixed method study with participants from the qualitative portion being drawn from the quantitative sample whose charactersitics were described as: “The majority of participants (72.5%) were 18–31 years old. The mean age of the most recent child was 11.4 months and the mean number of births was 2.5. Maternal educational status included (1) no formal education (18.4%); (2) completion of primary school (39.7%); and (3) completion of secondary school (37.1%). The most recent birth of most participants was attended by either a physician (39.4%) or a nurse (19.7%). The most recent births occurred in a public hospital (46.1%) or in a home (38.7%).”	Note: this was a mixed methods study. Only the qualitative results pertaining to this review are given here. *From abstract:* “Qualitative data revealed that dietary diversity may be low because (1) mothers often struggle to introduce complementary foods and (2) those that are traditionally introduced are not varied and primarily consist of grains and starches.” *Additional notes:* Qualitative data revealed several beliefs that may limit implementation of optimal IYCF practices. Women in some communities felt it was necessary to administer purgatives shortly after birth to cleanse the child’s body. A poor diet is believed to weaken breast milk, making it inadequate in terms of quality and/or unsatisfying to the child. Illness is thought to make breast milk “dirty” and unsafe for the child. In addition to these beliefs about breast milk, women view continued breastfeeding as a possible risk for infections among older children and thus prefer earlier weaning. Minimum dietary diversity achievement appears limited by difficulties mothers experience when complementary feedings and the low diversity of traditional, grain-based complementary feeding.	(2) (3) (4) (12) (19) (39) (51) (65)
12	Gretel H. Pelto, Margaret Armar-Klemesu Identifying interventions to help rural Kenyan mothers cope with food insecurity: results of a focused ethnographic study	Not stated	*From abstract:* “The results provide qualitative evidence about facilitators and constraints to IYC nutrition in the two geographical areas and document their inter-connections. We conclude with suggestions to consider 13 potential nutrition-sensitive interventions. The studies provide empirical ethnographic support for arguments concerning the importance of combining nutrition-specific and nutrition-sensitive interventions through a multi-sectoral, integrated approach to improve the nutrition of infants and young children in low-income, resource-constrained populations.” *Additional notes:* Not having enough money to buy food was commonly reported as a challenge associated with caring for their children. Shortage of water/lack of access to clean water was also a difficulty, both in taking time from mothers in water retrieval and in limiting food preparation. Collecting firewood and food storage limitations were also reported as issues impacting food preparation for young children. Mothers knew the importance of food and nutrition to their child’s health and development.	(37) (39) (40) (41) (42) (43) (44)
13	Alessandra N Bazzano, Richard A Oberhelman, Kaitlin Storck Potts, Leah D Taub, Chivorn Var What health service support do families need for optimal breastfeeding? An in-depth exploration of young infant feeding practices in Cambodia	Mothers’ ages ranged from 21 to 55. The range of education attainment was between 0 and 16 years. Ten mothers were housewives. Other mothers worked as a factory worker (*n* = 4), farmer (*n* = 3), or, sewer/weaver (*n* = 4), The rest of mothers’ occupation included small convenience store worker, clothes seller, nighttime guard (father), and high school teacher.	*From abstract:* “The results revealed knowledge and practice gaps in behavior that likely contribute to breastfeeding barriers, particularly in the areas of infant latch, milk production, feeding fre­quency, and the use of breast milk substitutes. The predominant theme identified in the research was a dearth of detailed information, advice, and counseling for mothers beyond the message to exclusively breastfeed for 6 months.” *Additional notes:* Women knew to practice exclusive breastfeeding for 6 months and reported following this behavior, however some mothers that reported this also said they used breastmilk substitutes on occasion. Some women described giving prelacteal feeds or waiting for the milk to come in prior to initiating breastfeeding. Some mothers gave water to newborns. Women reported that their own mothers, other female relatives or their midwives advised them on how to breastfeed. One mother reported that the midwife advised her to exclusively breastfeed but did not give her specific instructions to do so. Participants believed milk supply was related to the mother’s nutrition. Some felt they did not have enough milk and one was concerned about transferring illness to her newborn via breastmilk. Some felt that newborns required less milk or shorter feedings (or from just one breast) than older babies required. Some also did not want to rouse a sleepy newborn to feed. Many women had concerns that breastfeeding would impact their breast shape and size. They especially felt this could occur if they used both breasts during one feeding session. Positioning the newborn was also difficult for some mothers. Observations confirmed problems of poor latch, pain from suboptimal infant positioning and the practice of feeding from only one breast per session.	(1) (10) (11) (1) (19) (22) (25) (26) (28) (29) (33) (36) (37) (59) (62) (63)
14	Jennifer Burns, Jillian A. Emerson, Kimberly Amundson, Shannon Doocy, Laura E. Caulfield, Rolf D. W. Klemm A Qualitative Analysis of Barriers and Facilitators to Optimal Breastfeeding and Complementary Feeding Practices in South Kivu, Democratic Republic of Congo	The mean age of mothers was 25.8 years. Almost half of the mothers were literate (44%), however, around 28% had never attended school. Most of the mothers reported small businesses (47%) and agriculture (46%) as sources of income for their households.	*From abstract:* “Although breastfeeding was prevalent, few mothers engaged in optimal feeding practices. Barriers included poverty, high work burden, lack of decision-making power in the household, and perceived milk insufficiency. Health provider guidance and mothers’ motivation to breastfeed and feed nutrient-dense foods emerged as facilitators to optimal practices.” *Additional notes:* Many women recognized the benefits of colostrum for their newborn and gave it to them. Many said their mothers or grandmothers did not practice this and discarded the colostrum, but advice from health centers has changed this behavior. Common reasons for discontinuing exclusive breastfeeding included the feeling of insufficient milk, clogged milk duct, and the child being ill. These reason led to early introduction of complementary foods, water, or sugar water. The weather being hot was another reason cited for giving water to an infant less than 6 months old. Crying was considered an indication of the child being unsatisfied with breastmilk alone, so also led to early introduction of complementary foods like porridges. They were encouraged to continu this practice when giving other foods led to the baby stopping crying. Women who succeeded in exclusive breastfeeding for 6 months had encouragement during ANC visits. Mothers also reported that their own diets were important to the success of exclusive breastfeeding. Children’s diets (older than 6 months) have little diversity, and they eat what the family eats. Families with livestock do not tend to give the meat and milk available to their children as they prefer to sell these products in the market. The little meat the family eats is usually purchased, and milk is not commonly given to children as it is expensive.	(8) (10) (11) (18) (19) (22) (31) (32) (33) (36) (37) (39) (41) (45) (51) (63) (66)
*Grey Literature*				
15	Alive and Thrive-Ethiopia IYCF Practices, Beliefs, and Influences in Tigrey Region, Ethiopia	Not stated	*From summary:* “The findings from this study showed that although mothers have basic breastfeeding and complementary feeding information, visible gaps remain, such as not practicing exclusive breastfeeding, the existence of faulty traditional beliefs, and specific misconceptions about breastfeeding and complementary feeding. Among the major misconceptions is the belief of mothers that giving only breastmilk without adding fenugreek juice will expose the baby to intestinal worms. Beliefs also keep mothers from providing extra breastfeeding for a sick child. Some mothers reported the belief that encouraging suckling when the child is ill will only contribute to its illness. In Tigrey Region, mothers, community members, and HEWs alike stressed the unavailability of diverse foods and lack of resources to purchase foods as the major reasons for not following recommended complementary feeding practices. Lack of food was also mentioned in explaining why mothers introduce complementary foods later than 6 months of age, fail to offer children a variety of food types, and are unable to give children extra meals when they are sick or recovering from illness. The study identified misconceptions that affect complementary feeding habits. Mothers, community leaders, and even voluntary community health promoters (VCHPs) hold widespread beliefs that children cannot digest meat or other animal products, that children will choke on thick porridges, that extra food when a child is sick will contribute to illness, that children will refuse to eat during or after sickness, and that bottle feeding is more sanitary than using cups or hands to feed. Mothers and other community members, HEWs, VCHPs, and supervisors all expressed interest in learning about the recommended IYCF practices, as long as they trust that the person providing advice is properly trained and has reliable information. The findings from this study reveal that HEWs have reasonably better knowledge in the area of breastfeeding than in complementary feeding. Regarding their own knowledge and skills related to counseling, almost all of the HEWs are keen to improve those skills.”	(8) (20) (22) (33) (37) (39) (40) (41) (42) (48) (49) (50) (51) (53) (54) (55) (56) (57) (63) (65) (71) (72) (73)
16	Alive and Thrive IYCF Practices, Beliefs, and Influences in SNNP Region, Ethiopia	Not stated	*From summary:* This study indicates that breastfeeding is widely practiced in the research communities and is expected of all mothers and valued in the community for its contribution towards child growth and healthy development. Mothers in the study areas seem to know the expected breastfeeding requirements such as: on-demand feeding, frequency of breastfeeding, and the need to feed from both breasts. Yet exclusive breastfeeding is not widely practiced in the study communities. Mothers commonly introduce water, fenugreek, or linseed water as early as 2 months. In regards to complementary foods, the study indicates that the age for introduction of additional foods ranges from 2 months to 8 months, with a majority of mothers reporting that they had started giving foods at 6 months or earlier. Certain foods such as kale, enichila, banana, egg, pumpkin, carrot, and green vegetables are considered unsuitable and “too strong” for young children to digest before 1 year of age. Mothers consider meat in particular as too difficult to digest. Most mothers recommend meat for children above 1 or 2 years old. Mothers also expressed that they need to have a good income to buy or feed their children such foods. The study also revealed that HEWs feel they have a need to learn more about child feeding in order to effectively counsel mothers on IYCF. Several pointed out that they need more detailed information about additional foods, types of foods, and best approaches for preparing food for young children, with follow-up mechanism.	(5) (8) (10) (11) (18) (22) (27) (39) (41) (51) (52) (53) (55) (56) (62) (63) (65) (70) (71) (72)
17	United States Agency for International Development Formative assessment of infant and young child feeding practices at the community level in Zambia	Not stated	Breastfeeding is recognized as extremely important by mothers and other members of the community and remains the first choice for infant feeding. There is widespread awareness about the message of exclusive breastfeeding for six months, and generally mothers, fathers and grandmothers expressed their support for the practice. However, in reality many respondents expressed doubts as to whether breastmilk was really sufficient during the first six months and whether mothers could produce enough breastmilk to meet their child’s needs. In particular, families tended to introduce water before six months due to their perception that infants gesture to indicate their needs—and the belief that not responding is cruel to the child. And it is traditional to introduce water and watery foods early. When women are away from their infants, most respondents did not feel it was acceptable to express breastmilk. Many respondents strongly oppose it because it is considered untraditional, unhygienic, and unnecessary if the mother can stay close to her child. Multiple respondents commented that when mothers have an inadequate diet, they do not produce enough breastmilk, and need to introduce foods and liquids before 6 months of age, or must wean their child from breastmilk. The study identified a number of factors that represent barriers to the uptake of recommended infant and young child feeding practices. Respondents noted that cultural beliefs, traditions, and lack of knowledge about the nutritional needs of young children are barriers (e.g., most women give thin unenriched porridges as complementary foods). Many respondents identified lack of food and inability to afford food as a major reason for not providing appropriate foods to young children. Mothers did not cite grandmothers and fathers as important influencers of IYCF. The media was cited as a source of information on IYCF.	(8) (10) (11) (18) (19) (20) (22) (31) (32) (33) (34) (37) (39) (41) (62) (63) (65)
18	United States Agency for International Development Formative assessment of infant and young child feeding practices-federal capital territory, Nigeria	Not stated	Prelacteal feeding of water and sometimes water with herbs was widespread. There were different beliefs regarding colostrum but some believed (with influence by the grandmother) that it was bad and dangerous for the infant. A traditional practice is to give herbal infusions in water to treat and prevent illness, from the time of birth. Being away from baby inhibited breastfeeding, and often results in introduction of water. Many of the women are farmers and it is difficult to breastfeed when they are working on the farm. Breastmilk is not believed to be sufficient in either water or sustenance for babies 3 months and older, so thin gruels are introduced at this age. Some women believed that a mother with HIV could not breastfeed her baby. A sick child with a lack of appetite requires “forced feeding” but some felt concern that this might make the child choke (child sickness impacts child feeding). Maternal nutrition is recognized as important for pregnant and lactating women, and inadequate nutrition leads to inadequate milk production, but there was a lack of consensus of what foods are best for mothers and affordability may impact their ability to attain good foods. Fathers were supportive of the women and children achieving good nutrition by letting them eat first.	(7) (17) (18) (19) (20) (22) (27) (32) (33) (37) (47) (50) (62) (63) (65) (70) (72) (73)
19	United States Agency for International Development Consulting with caregivers–formative research to determine the barriers and facilitators to optimal infant and young child feeding in three regions of Malawi	Mothers in both phases were young, with more than half in each sample less than 25 years old. The overall literacy rate of the women in both samples (63.3 percent in Phase 1 and 69 percent in Phase 2) was slightly less than the national literacy average of 70 percent, 19 and in both samples, there was a wide variation in years of schooling, with approximately one-fifth to one-quarter of each group having no schooling at all and another quarter having more than six years of schooling.	Crying after feeding influenced the mother or other family members to give the baby other foods or liquids, assuming that the breastmilk was inadequate to satiate the baby’s hunger. Giving water during hot times of the year was thought to be necessary by some mothers, interfering with exclusive breastfeeding. Mothers did not know that breastmilk alone was acceptable for infants less than 6 months; once they were told this and tried it many were successful at transitioning to the improved practice. Observations identified two suboptimal breastfeeding practices: breastfeeds of very short duration, and breastfeeding from just one breast. When mothers transitions to improved practices, positive outcomes reinforced this behavior, including less crying and better sleep for babies, which give mothers more time for household chores, babies took more milk and did not have an upset stomach. Other family members support also facilitated good practice, for example by identifying that longer duration of breastfeeding indicated mother was showing more love to the child. Fear of HIV transmission was indicated as a reason for stopping continued breastfeeding early, along with having another pregnancy. The study also found that using breastfeeding to prevent pregnancy was also a facilitator of continued breastfeeding, but that mothers need counseling on the use of this method. Families given infants 6 to 8 months thin/watery porridges because they think this is the only food this age child can swallow and digest.	(8) (10) (11) (18) (21) (31) (34) (38) (41) (51) (53) (55) (63) (65) (66) (73)
20	Academy for Educational Development (AED)/ Alive and Thrive Ethiopia Country Office Initial Insight Mining and Pretest Research for Alive and Thrive Ethiopia	Not stated	Breastfeeding is common, but exclusive breastfeeding remains difficult for most mothers are they are responsible for a lot of work both in and out of the home. They are very busy and have limited access to information, do not have time to prepare special foods for children or exclusively breastfeed for six months (especially for mothers who work in the fields). Overall, there is a lack of awareness about nutritional requirements for infants and young children. Adding oil to baby food is common, and meat is considered healthy but not given to children under 2 years because it is thought babies cannot digest meat. Thick porridge is also considered to be hard to digest, so thin porridge is given to young children. Eggs are sold in the market rather than given to children because they are expensive; the same applies to milk—if they have a cow they sell the milk in market, and many cannot afford a cow or goat. There was a lack of awareness that different foods provided different nutrients, or that nutrition was related to mental and physical development. Respondents were not aware of the importance of the first two years of life. In most locations, there is lack of availability of several food categories, especially meat and fish, and they are expensive when they are available.	(14) (21) (22) (32) (33) (39) (41) (45) (51) (55)
21	United States Agency for International Development Engaging grandmothers and men in infant and young child feeing and maternal nutrition—Report of a formative assessment in Eastern and Western Kenya	Not stated	Although infant and young child care are considered primarily the mother’s realm, the study found that cultural beliefs also play an important role and grandmothers are largely responsible for propagating these beliefs. Grandmothers influence the early introduction of other foods, as a response to babies crying, indicating to them that breastmilk alone is insufficient. Feeding porridge to young babies also induces longer sleep sessions, which is a motivation for mothers to interrupt exclusive breastfeeding since it allows them to do more other activities. Some mothers’ negative connotation regarding exclusive breastfeeding was due to the perceptions that those who exclusively breastfed for 6 months were HIV positive. Reasons for early cessation of breastfeeding included pregnancy, doctor’s advice, baby refusing to eat other foods, and reduction in breastmilk supply. Mothers had minimal awareness of best complementary feeding practices. Resulting sub-optimal practices included early introduction of complementary foods, low quality foods in terms of energy and nutrient content, and inadequate feeding frequency. Foods given to young children are limited to what the household can afford (they eat modified family foods usually). Heavy workloads of the primary caregiver impacts complementary feeding behaviors. Force-feeding was reported as a common practice in Western Province. Some caregivers are only able to cook food, usually porridge, for the baby once a day and then store it in a flask the rest of the day, increasing the chance of bacterial growth. Grandmothers in Eastern Province believed that meat should not be given the children before 2 years of age, impacting the quality of diets of children. The study found that grandmothers and men have inadequate knowledge of recommended IYCF practices. Men listen to the advice of their mothers (the child’s grandmother) more than their wives regarding infant and childcare and feeding, since they are considered more experienced. Men may not be clear about their role in IYCF besides providing food. Men are interested in obtaining more information on IYCF but there are no forums to do so besides general community-level initiatives like flyers, radio programs, or messages from church. Men want to learn about best practices form trained experts, and trust those who they perceived as more knowledgeable on the topic. The majority of fathers did not believe exclusive breastfeeding for 6 months would be feasible due to the mother’s many responsibilities that require separation from the infant. Men agreed that they must provide mothers with adequate food to allow them to breastfeed successfully. Grandmothers are highly regarded as knowledgeable and experienced caretakers in the communities and so play a large role in infant and child caregiving and feeding. They also often provide financial support to their son’s young families. Grandmothers are the main alternative caregivers when mothers are away. They are also central in decision-making regarding food preparation and child feeding, along with other important activities like sick-child care.	(8) (10) (11) (16) (18) (19) (20) (21) (27) (33) (37) (39) (51) (58) (65) (71) (73)
22	United States Agency for International Development Ghana promotion of complementary feeding practices project—baseline survey report	The majority of respondents (42.2%) were between the ages of 22 and 29 years. The median age was 28 years. More than half of the respondents (56.3%) in the nine districts had little or no education (primary school or less). Only one-third had completed basic education (middle school or junior high school). District-specific data showed a similar result in only three of the districts: Sene, Sunyani West, and Kintampo South having respondents with tertiary level education. The majority were peasant farmers, including a few fishermen (55.2%); followed by petty traders (13.7%) and artisans (12.2%). A little more than 10% (11.5%) reported that they were unemployed. About 85% of the respondents were married. Only 7% of participants were single (never married), and 6.3% were cohabiting. The majority of the study participants reported Christianity as their religion (81.5%). This was followed by Islam (11.5%), with a small percentage being traditionalists.	*Note:* The majority of findings presented are from the quantitative portions of this study, so are not relevant for this review. Mothers and fathers who believed exclusive breastfeeding was good for baby < 6 months said that it helps a baby grow and protects them from breastfeeding. Those who did not believe exclusive breastfeeding was best said that this was because a baby needs more than just breastmilk to satisfy them (they also need water).	(18) (33) (65) (70)
23	Alive and Thrive Bangladesh Perceptions, practices, and promotion of infant and young child feeding—results and program implications of assessments in Bangladesh	Not stated	*From summary:* “Delayed initiation of breastfeeding, pre-lacteal feeding, and lack of exclusive breastfeeding were found to be common largely due to a lack of understanding of their importance and confusion about colostrum feeding as well as the definition of EBF. Also, mothers lack support for appropriate infant feeding after delivery. The perception that baby’s crying indicates insufficient breastmilk and that insufficient breastmilk is caused by poor maternal diets is widespread and a major barrier to EBF.” *Additional notes:* Perceptions of what foods should be given for complementary feeding of children 6 to 24 months differ from the recommended practices. There is a lack of diversity of type of food available to give children, especially animal sourced foods. Child’s poor appetite causes them to reject foods, inhibiting optimal feeding. Mother’s time constraints and lack of knowledge of appropriate complementary feeding were viewed as larger barriers than limited food availability. Mothers lack knowledge of how to make family foods appropriate for young babies and therefore do not give them meat because it is presumed to be not appropriate for them.	(10) (11) (19) (24) (39) (46) (48) (51) (55)

## Appendix C

Details of CERQual qualitative evidence profile of barriers and facilitators to recommended breastfeeding and complementary feeding practices.

**Table:**

Finding No.	Review Finding	Studies Contributing to the Review Finding	Assessment of Methodological Limitations	Assessment of Relevance	Assessment of Coherence	Assessment of Adequacy	Overall CERQual Assessment of Confidence	Explanation of Judgement
Barriers								
Barriers to Recommended Breastfeeding								
Key Theme: *Beliefs and Perceptions*								
1	Breastfeeding deforms breasts	1; 4; 13	Minor methodological limitations (the authors do not clarify potential bias)	Minor concerns about relevance (the finding comes from studies in three settings across different locales: Cambodia, Dominican Republic, and Ghana)	Minor concerns about coherence (data consistent within and across both studies)	No concern about adequacy of data (the authors provide rich data)	High confidence	This finding was graded as high confidence because of few concerns about coherence and relevance and moderate methodological limitations.
2	Breastfeeding for more than one year makes children susceptible to infection	11	Minor methodological limitations (the authors do not state potential bias)	NA	NA	Minor concern about adequacy (the authors offer moderately rich data overall)	NA	NA
3	Breastmilk is bad because a mother is mad	11	Minor methodological limitations (the authors do not state potential bias)	NA	NA	Minor concern about adequacy (the authors offer moderately rich data overall)	NA	NA
4	Breastmilk is dirty because a mother has an illness	11	Minor methodological limitations (the authors do not state potential bias)	NA	NA	Minor concern about adequacy (the authors offer moderately rich data overall)	NA	NA
5	Breastmilk is not enough for a small baby	8; 16	Substantial methodological limitations (both studies do not explain the sampling method, eligibility criteria and potential bias)	Moderate concerns about relevance (partial relevance, as the studies were from only two settings: Indonesia and Ethiopia)	Minor concerns about coherence (data consistent within and across both studies)	Moderate concerns about adequacy (only two studies, one provides rich data, but the other one offers thin data)	Low confidence	This finding was graded as low confidence because of moderate concerns about relevance and adequacy of data and substantial methodological limitations.
6	When baby belches on the breast during breastfeeding the mother can develop swollen breasts	4	Moderate methodological limitations (the study does not state analysis methods and potential bias)	NA	NA	No concern about adequacy of data (the authors provide rich data)	NA	NA
7	Colostrum is not healthy for baby, so mothers discard colostrum	3; 7; 10; 18	Moderate methodological limitations (all studies do not discuss potential bias; two studies do not state analysis methods; one does not say eligibility criteria)	Minor concerns about relevance (studies of breastfeeding from four countries: Ghana, Vietnam, Haiti and Nigeria)	Minor concerns about coherence (data reasonably consistent within and across all studies)	Minor concerns about adequacy (three studies offer rich data overall while one study shows thin data)	Moderate confidence	This finding was graded as moderate confidence because of minor concerns regarding relevance, coherence, and adequacy and moderate methodological limitations.
8	Baby remains hungry after breastfeeding (e.g., a baby is fussy even after breastfeeding)	5; 6; 8; 9; 14; 15; 16; 17; 19; 21	Moderate methodological limitations (all studies with moderate methodological limitations)	Minor concerns about relevance (the review finding is applicable from four countries: Colombia, Ethiopia, Zambia and Kenya)	Minor concerns about coherence (data reasonably consistent within and across all studies)	Moderate concerns about adequacy (two studies provide rich data, but three studies offer only thin data)	Moderate confidence	This finding was graded as moderate confidence because of moderate concerns regarding methodological limitations and adequacy of data and minor concerns about relevance and coherence.
9	Formula milk can make a baby healthier	8	Moderate methodological limitations (the authors do not explain the sampling method, eligibility criteria and potential bias)	NA	NA	No concern about adequacy (the authors provide rich data)	NA	NA
10	Mothers do not produce sufficient breastmilk	3; 4; 5; 6; 7; 8; 9; 13; 14; 16; 17; 19; 21; 23	Moderate methodological limitations (all studies with moderate methodological limitations)	Minor concerns about relevance (the review finding is universal in 13 countries: Cambodia, Democratic Republic of Congo, Ghana, Colombia, Bangladesh, Vietnam, Indonesia, Laos PDR, Ethiopia, Zambia, Malawi, Kenya and Bangladesh)	Minor concerns about coherence (data reasonably consistent within and across all studies)	Minor concerns about adequacy (the majority of the studies provide rich data. Only one study offers thin data)	High confidence	This finding was graded as high confidence because of minor concerns about relevance, coherence and adequacy and moderate methodological limitations.
11	A baby is thirsty	2; 7; 9; 13; 14; 16; 17; 19; 21; 23	Moderate methodological limitations (all studies with moderate methodological limitations)	Minor concerns about relevance (the review finding is relevant across nine countries: Cambodia, Democratic Republic of Congo, Tanzania, Vietnam, Lao PDR, Ethiopia, Zambia, Malawi, Kenya and Bangladesh)	Minor concerns about coherence (data reasonably consistent within and across all studies)	Moderate concerns about adequacy (six studies provide moderately rich data overall, but four studies offer thin data)	Moderate confidence	This finding was graded as moderate confidence because of moderate concerns regarding methodological limitations and adequacy of data and minor concerns about relevance and coherence.
12	Purgative cleans a baby	11	Minor methodological limitations (the authors do not state potential bias)	NA	NA	Substantial concerns about adequacy (only short quotes are available)	NA	NA
13	Arrival of breastmilk is late	3	Moderate methodological limitations (the authors do not explain analysis methods and potential bias)	NA	NA	No concern about adequacy of data (the authors provide rich data)	NA	NA
14	Giving fenugreek and traditional herbs to newborns or young babies is a traditional practice	20	Moderate methodological limitations (the authors do not state sampling, analysis method and potential bias.	NA	NA	Minor concerns about adequacy (the authors offer only thin data)	NA	NA
15	Mothers do not have confidence in sufficient production of breast milk	5	Moderate methodological limitations (the authors do not state sampling and potential bias)	NA	NA	No concern about adequacy of data (the study provides rich data)	NA	NA
16	A baby still cries even after a mother breastfeeds	21	Moderate methodological limitations (the authors do not state sampling and potential bias)	NA	NA	No concern about adequacy of data (the authors provide rich data)	NA	NA
17	Herbal infusions in water prevents a baby from diseases.	18	Moderate methodological limitations (the authors do not state sampling and potential bias)	NA	NA	No concern about adequacy of data (the study shows rich data)	NA	NA
18	Traditional practices do not require exclusive breastfeeding (e.g., pre-lacteal feeds, giving water)	2; 7; 14; 16; 17; 18; 19; 21; 22	Moderate methodological limitations (all studies with moderate methodological limitations)	Minor concerns about relevance (the review finding is relevant across nine countries: Democratic Republic of Congo, Tanzania, Vietnam, Ethiopia, Zambia, Nigeria, Malawi, Kenya and Ghana)	Moderate concerns about coherence (data in four studies lack a convincing explanation for the patterns found in these data while data in other five studies relatively are consistent within and across studies)	Moderate concerns about adequacy of data (three studies with thin data. Six studies with rich data).	Moderate confidence	This finding was graded as moderate confidence because of minor concerns about relevance and moderate concerns regarding methodological limitations and coherence and adequacy of data.
19	Mothers attribute their inability to produce enough breastmilk to not eating well	11; 13; 14; 17; 18; 21; 23	Moderate methodological limitations (five studies with moderate methodological limitations and one study with minor methodological limitations)	Minor concerns about relevance (the review finding is universal in seven countries: Democratic Republic of Congo, Zambia, Nigeria, Kenya, Haiti, Cambodia and Bangladesh)	Minor concerns about coherence (data reasonably consistent within and across all studies)	Minor concerns about adequacy of data (all data provides moderately rich data overall)	High confidence	This finding was graded as high confidence because of minor concerns about relevance, coherence and adequacy and moderate methodological limitations.
20	Pregnant women should not breastfeed (Mothers become pregnant before the child is 2 years old)	15; 17; 18; 21	Moderate methodological limitations (all studies with moderate methodological limitations)	Minor concerns about relevance (the review finding is relevant across four countries: Ethiopia, Zambia, Nigeria and Kenya)	Minor concerns about coherence (data reasonably consistent within and across all studies)	Moderate concerns about adequacy of data (two studies offer relatively rich data, but other studies show only thin data)	Moderate confidence	This finding was graded as moderate confidence because of minor concerns about relevance, coherence and moderate concerns about methodological limitations and adequacy of data.
Key Theme: *Lack of Support from Families, Health Workers (HWs) and Others*								
21	Family members do not support mothers to breastfeed	4; 19; 20; 21	Moderate methodological limitations (all studies with moderate methodological limitations)	Minor concerns about relevance (the review finding is relevant across four countries: Ghana, Kenya, Malawi and Ethiopia)	Minor concerns about coherence (data reasonably consistent within and across all studies)	Substantial concerns about adequacy (three studies provide poor data. Only one study offers rich data).	Moderate confidence	This finding was graded as moderate confidence because of minor concerns about coherence and relevance; moderate methodological limitations; and substantial concerns about adequacy.
22	Mothers do not have enough time to breastfeed their infants due to their work and short maternity leave	4; 6; 7; 8; 9; 13; 14; 15; 16; 17; 18; 20	Moderate methodological limitations (all studies with moderate methodological limitations)	Minor concerns about relevance (the review finding is relevant across ten countries: Ghana, Vietnam, Bangladesh, Indonesia, Lao PDR, Cambodia, Democratic Republic of Congo, Ethiopia, Zambia and Nigeria)	Minor concerns about coherence (data reasonably consistent within and across all studies)	Moderate concerns about adequacy (the majority of the studies provide moderately rich data overall, but three studies offer thin data)	Moderate confidence	This finding was graded as moderate confidence because of minor concerns about relevance and moderate concerns regarding methodological limitations and coherence and adequacy of data.
23	HWs believe mothers do not produce enough breastmilk	3	Moderate methodological limitations (the authors do not explain analysis methods and potential bias)	NA	NA	No concern about adequacy of data (the authors provide rich data)	NA	NA
24	HWs do not have adequate knowledge and skills to help mothers	2; 9; 23	Moderate methodological limitations (all studies have moderate methodological limitations)	Moderate concerns about relevance (partial relevance, as the studies were from only two settings: Tanzania, Laos and Bangladesh)	Minor concerns about coherence (data consistent within and across both studies)	Minor concerns about adequacy (all studies provide rich data)	Moderate confidence	This finding was graded as moderate confidence because of minor concerns about coherence and adequacy; moderate concern about relevance and methodological limitations.
25	Mothers do not receive any advice from HWs	3; 9; 10; 13	Minor methodological limitations (two studies with minor limitations and the other studies with moderate limitations)	Minor concerns about relevance (the review finding is universal across four countries: Ghana, Laos PDR, Haiti and Cambodia)	Minor concerns about coherence (data consistent within and across both studies)	Minor concerns about adequacy (three studies provide rich data while only one study offers thin data)	High confidence	This finding was graded as high confidence because of few concerns about methodological limitations, relevance, coherence and adequacy.
26	Media has an impact on use of formula milk (and encourages it by instilling the belief that formula is healthy for baby)	7; 8; 13	Moderate methodological limitations (all studies do not consider potential bias. One study do not describe sampling methods)	Minor concerns about relevance (the review finding is relevant across three countries in Asia: Indonesia, Cambodia and Vietnam)	Minor concerns about coherence (data consistent within and across both studies)	No concern about adequacy of data (both studies offer rich data)	High confidence	This finding was graded as high confidence because of few concerns about coherence and relevance and moderate methodological limitations.
27	Traditional cultural beliefs of female family members (e.g., grandmothers) influence breastfeeding	7; 16; 18; 21	Moderate methodological limitations (all studies with moderate methodological limitations)	Minor concerns about relevance (the review finding is relevant across four countries: Vietnam, Ethiopia, Kenya and Nigeria)	Minor concerns about coherence (data consistent within and across both studies)	Moderate concerns about adequacy (two studies show poor data. The other studies provide moderately rich data overall)	Moderate confidence	This finding was graded as moderate confidence because of minor concerns about coherence and relevance; moderate concern about methodological limitations and adequacy.
28	Suggestions from families, peers or close neighbors influence breastfeeding	8; 9; 13	Moderate methodological limitations (two studies do not consider potential bias. One does not state sampling. One does not explain analysis method)	Minor concerns about relevance (partial relevance, as the studies were from three settings: Cambodia, Indonesia and Laos)	Minor concerns about coherence (data consistent within and across both studies)	No concern about adequacy of data (all studies offer rich data)	High confidence	This finding was graded as high confidence because of adequate data, minor concerns about coherence and relevance and moderate concerns about methodological limitations
Key Theme: *Infant-Specific Factors*								
29	A baby stops sucking milk	9; 13	Moderate methodological limitations (one study do not explain potential bias. The other one states response bias and does not explain analysis methods)	Moderate concerns about relevance (partial relevance, as the studies were from only two settings: Cambodia and Laos)	Minor concerns about coherence (data consistent within and across both studies)	No concern about adequacy of data (both studies offer rich data)	Moderate confidence	This finding was graded as moderate confidence because of minor concerns about coherence, moderate concern about relevance and methodological limitations
30	Delayed breastfeeding initiation occurs with infants with respiratory problems	10	Minor methodological limitations (the study has some missing data)	NA	NA	No concern about adequacy of data (rich data)	NA	NA
31	Baby has a sickness	4; 10; 14; 17; 19	Moderate methodological limitations (the majority of the studies have moderate methodological limitations. Only one study has minor limitations.	Minor concerns about relevance (the review finding is relevant across five countries: Ghana, Haiti, Democratic Republic of Congo, Nigeria and Malawi)	Minor concerns about coherence (data consistent within and across both studies)	Substantial concerns about adequacy (only one study offers rich data. Others show thin data)	Moderate confidence	This finding was graded as moderate confidence because of substantial concerns about adequacy; moderate methodological limitations; and minor concerns regarding coherence and relevance.
Key Theme: *Mothers’ Knowledge*								
32	Mothers do not have knowledge about colostrum and breastfeeding initiation	3; 7; 8; 14; 17; 18; 20	Moderate methodological limitations (only one study with minor methodological limitations. Other studies with moderate methodological limitations)	Minor concerns about relevance (the review finding is relevant across seven countries: Ghana, Vietnam, Indonesia, Democratic Republic of Congo, Zambia, Nigeria and Ethiopia)	Minor concerns about coherence (data consistent within and across both studies)	Substantial concerns about adequacy (only three studies provide moderately rich data overall. Others offer thin data)	Moderate confidence	This finding was graded as moderate confidence because of substantial concerns about adequacy; moderate methodological limitations; and minor concerns regarding coherence and relevance
33	Mothers do not have knowledge about breastfeeding	8; 9; 10; 13; 14; 15; 17; 18; 20; 21; 22	Moderate methodological limitations (only one study with minor methodological limitations. Other studies with moderate methodological limitations)	Minor concerns about relevance (the review finding is relevant across ten countries: Indonesia, Lao PDR, Haiti, Cambodia, Democratic Republic of Congo, Zambia, Nigeria, Ethiopia, Kenya and Ghana)	Moderate concerns about coherence (three studies lack a convincing explanation. Data in other studies consistent within and across all studies)	Moderate concerns about adequacy (three studies provide poor data. Other studies offer moderately rich data overall)	Moderate confidence	This finding was graded as moderate confidence because of moderate concerns regarding methodology, coherence, adequacy and minor concerns about relevance.
34	Mothers do not recognize between baby feeding cues and behaviors	8; 17; 19	Moderate methodological limitations (all studies with moderate methodological limitations)	Moderate concerns about relevance (the review finding is found only in three settings: Indonesia, Zambia and Malawi)	Minor concerns about coherence (data consistent within and across both studies)	Minor concerns about adequacy (all studies provide moderately rich data)	Moderate confidence	This finding was graded as moderate confidence because of moderate concerns about methodology, relevance, coherence and minor concerns about adequacy.
35	Mothers do not have education	2	Moderate methodological limitations (the authors do not explain sampling and potential bias)	NA	NA	Minor concern about adequacy of data (the authors provide moderately rich data overall)	NA	NA
Key Theme: *Mother-Specific Factors*								
36	Mothers have breast and nipple problems	4; 5; 7; 9; 13; 14	Moderate methodological limitations (all studies with moderate methodological limitations)	Minor concerns about relevance (the review finding is relevant across seven countries: Ghana, Colombia, Vietnam, Lao PDR, Cambodia and Democratic Republic of Congo)	Minor concerns about coherence (data consistent within and across both studies)	Minor concern about adequacy of data (the majority of the studies provide moderately rich data overall, but one study shows only thin data)	High confidence	This finding was graded as high confidence because of minor concerns regarding relevance, coherence, adequacy and moderate methodological limitations.
37	Mothers have a sickness and/or died	4; 9; 12; 13; 14; 15; 17; 18; 21	Moderate methodological limitations (the most studies with moderate methodological limitations except one study with minor limitations)	Minor concerns about relevance (the review finding is relevant across seven countries: Ghana, Lao PDR, Kenya, Cambodia, Democratic Republic of Congo, Ethiopia, Zambia, and Nigeria)	Minor concerns about coherence (data consistent within and across both studies)	Substantial concerns about adequacy (only three studies show rich data, other studies provide only thin data)	Moderate confidence	This finding was graded as moderate confidence because of substantial concerns about adequacy of data, moderate methodological limitations and minor concerns regarding relevance and coherence.
38	Breastfeeding initiation is delayed due to pregnancy induced hypertension and other complications after childbirth (performance of post-birth activities)	3; 10; 19	Minor methodological limitations (two studies with minor concerns and one study with moderate concerns)	Moderate concerns about relevance (the review finding is found in only three studies: Chana, Haiti and Malawi)	Minor concerns about coherence (data consistent within and across both studies)	Substantial concerns about adequacy (all studies show poor data).	Moderate confidence	This finding was graded as moderate confidence because of substantial concerns about adequacy, moderate concerns about relevance and minor concerns about coherence and methodological limitations.
Barriers to Complementary Feeding								
Key Theme: *Food Security and Social and Cultural Factors*								
39	Mothers and families do not have enough money to buy foods	11; 12; 14; 15; 16; 17; 20; 21; 23	Moderate methodological limitations (seven studies with moderate methodological limitations and one study with minor limitations)	Minor concerns about relevance (the review finding is relevant across seven countries: Haiti, Kenya, Democratic Republic of Congo, Ethiopia, Zambia and Bangladesh)	Minor concerns about coherence (data consistent within and across both studies)	Minor concerns about adequacy (all studies provide moderately rich data overall except one study with thin data)	High confidence	This finding was graded as high confidence because of minor concerns about relevance, coherence and adequacy; and moderate methodological limitations.
40	In periods of food insecurity, especially during drought, the purchase of higher quality foods are replaced with cheaper, less nourishing foods	12; 15	Moderate methodological limitations (both studies do not consider potential bias. One study does not state analysis methods).	Moderate concerns about relevance (partial relevance, as the studies were from only two settings: Kenya and Ethiopia)	Minor concerns about coherence (data consistent within and across both studies)	No concern about adequacy of data (both studies provide rich data)	Moderate confidence	This finding was graded as moderate confidence because of minor concerns about coherence, moderate concern about relevance and methodological limitations
41	There are shortages of diverse foods to meet nutritional needs	12; 14; 15; 16; 17; 19; 20	Moderate methodological limitations (the majority of studies with moderate methodological limitations except with one study with minor concerns)	Minor concerns about relevance (the review finding is relevant across countries: Kenya, Democratic Republic of Congo, Ethiopia, Zambia and Malawi)	Minor concerns about coherence (data consistent within and across both studies)	Minor concerns about adequacy (all studies provide moderately rich data overall)	High confidence	This finding was graded as high confidence because of minor concerns about relevance, coherence and adequacy; and moderate methodological limitations.
42	There is no piped water in the houses for cooking	12; 15	Moderate methodological limitations (both studies do not consider potential bias. One study does not state analysis methods).	Moderate concerns about relevance (partial relevance, as the studies were from only two settings: Kenya and Ethiopia)	Minor concerns about coherence (data consistent within and across both studies)	No concern about adequacy of data (both studies provide rich data)	Moderate confidence	This finding was graded as moderate confidence because of minor concerns about coherence, moderate concern about relevance and methodological limitations
43	Some foods are only available seasonally	12	Minor methodological limitations (the authors do not clarify potential bias)	NA	NA	Minor concern about adequacy of data (the study offers moderately rich data overall)	NA	NA
44	Mothers prepare foods early in the day and then store it to give to their children later.	12	Minor methodological limitations (the authors do not clarify potential bias)	NA	NA	No concern about adequacy of data (the study provides rich data)	NA	NA
45	Mothers do not have financial decision-making power	14; 20	Moderate methodological limitations (both studies do not consider potential bias and analysis methods. One study does not describe sampling)	Moderate concerns about relevance (partial relevance, as the studies were from only two settings: Democratic Republic of Congo and Ethiopia)	Minor concerns about coherence (data consistent within and across both studies)	No concern about adequacy of data (both studies offer rich data)	Moderate confidence	This finding was graded as moderate confidence because of minor concerns about coherence, moderate concern about relevance and methodological limitations
Key Theme: *Infant-Specific Factors*								
46	Infants dislike animal source foods	6; 23	Moderate methodological limitations (both studies do not consider potential bias and analysis methods.)	Moderate concerns about relevance (partial relevance, as the studies were from only one country: Bangladesh)	Minor concerns about coherence (data consistent within and across both studies)	Substantial concern about adequacy (both studies provide thin data)	Low confidence	This finding was graded as low confidence because of moderate concerns about methodology and relevance; and substantial concerns about adequacy of data.
47	Infants do not have appetite to eat animal source foods	6; 18	Moderate methodological limitations (the authors in both studies do not consider potential bias. One study does not describe sampling. The other one does not explain analysis methods.)	Moderate concerns about relevance (partial relevance, as the studies were from only two settings: Bangladesh and Nigeria)	Minor concerns about coherence (data consistent within and across both studies)	Moderate concern about adequacy (One study provides rich data. The other one offers only thin data)	Moderate confidence	This finding was graded as moderate confidence because of moderate concerns regarding methodological limitations, adequacy of data, relevance and minor concerns about coherence.
48	Infants refuse to eat and or spit out of foods	6; 15; 23	Moderate methodological limitations (all studies with moderate methodological limitations)	Moderate concerns about relevance (partial relevance, as the studies were from only two settings: Bangladesh and Ethiopia)	Moderate concern about coherence (data partially consistent within and across both studies)	Substantial concerns about adequacy (all studies offer thin data)	Low confidence	This finding was graded as low confidence because of moderate concerns about relevance and coherence and substantial concerns about adequacy of data and methodology.
49	Infants vomit when animal source foods are offered	6; 15	Substantial methodological limitations (the authors in both studies do not consider potential bias and analysis methods. One study does not describe sampling.)	Moderate concerns about relevance (partial relevance, as the studies were from only two settings: Bangladesh and Ethiopia)	Moderate concern about coherence (data partially consistent within and across both studies)	Substantial concerns about adequacy (only two studies, both offering thin data)	Low confidence	This finding was graded as low confidence because of moderate concerns about relevance and coherence and substantial concerns about adequacy of data and methodology.
50	Infants have a sickness	15; 18	Moderate methodological limitations (the authors in both studies do not consider potential bias and analysis methods.)	Moderate concerns about relevance (partial relevance, as the studies were from only two settings: Nigeria and Ethiopia)	Minor concerns about coherence (data consistent within and across both studies)	No concern about adequacy of data (both studies offer rich data)	Moderate confidence	This finding was graded as moderate confidence because of moderate concerns about methods and methodology and minor concerns about coherence.
Key Theme: *Family-Specific Actors*								
51	Mothers and other family members do not have knowledge about complementary feeding	6; 11; 14; 15; 16; 19; 20; 21; 23	Moderate methodological limitations (all studies with moderate methodological limitations)	Minor concerns about relevance (the review finding is relevant in six countries: Bangladesh, Haiti, Democratic Republic of Congo, Ethiopia, Malawi and Kenya)	Minor concerns about coherence (data consistent within and across both studies)	Minor concerns about adequacy (two studies offer only thin data, but other studies provide rich data overall)	High confidence	This finding was graded as high confidence because of minor concerns about relevance, coherence and adequacy; and moderate methodological limitations.
52	Mothers are concerned about over-feeding their child	16	Moderate methodological limitations (the study does not consider potential bias and analysis methods)	NA	NA	No concern about adequacy of data (the study provides rich data)	NA	NA
53	Mothers and family members have fear that child may choke on certain solid foods	15; 16; 19	Moderate methodological limitations (all studies do not consider potential bias. Two studies do not explain analysis methods)	Moderate concerns about relevance (partial relevance, as the studies were from only three settings: Malawi and SNNP and Tigrey Ethiopia)	Minor concerns about coherence (data consistent within and across both studies)	Moderate concern about adequacy of data (one study provides moderately rich data overall, but other studies offer only thin data)	Moderate confidence	This finding was graded as moderate confidence because of moderate concerns about methodology, adequacy of data, relevance and minor concern regarding coherence.
54	Mothers and other family members are not willing to prepare non-fasting foods for children	15	Moderate methodological limitations (the study does not consider potential bias and analysis methods)	NA	NA	Moderate concern about adequacy of data (the study provides only thin data)	NA	NA
55	Thick porridge is believed to be difficult for young children to digest and thus avoided	15; 16; 19; 20; 23	Moderate methodological limitations (all studies with moderate methodological limitations)	Moderate concerns about relevance (partial relevance, as the studies were from only three countries: Malawi, Ethiopia and Bangladesh)	Minor concerns about coherence (data consistent within and across both studies)	Moderate concern about adequacy of data (one study provides moderately rich data overall, but other studies offer only thin data)	Moderate confidence	This finding was graded as moderate confidence because of moderate concerns about methodology, adequacy of data, relevance and minor concern regarding coherence.
56	Mothers and family members do not have enough time to cook for their child	15; 16	Moderate methodological limitations (both studies do not consider potential bias and analysis methods)	Moderate concerns about relevance (partial relevance, as the studies were from only two settings: Tigrey and SNNP Ethiopia)	Minor concerns about coherence (data consistent within and across both studies)	Minor concern about adequacy of data (both studies provide moderately rich data overall)	Moderate confidence	This finding was graded as moderate confidence because of moderate concerns about methodology and relevance and minor concerns regarding coherence and adequacy of data.
Cross-Cutting Barriers								
57	Inability of HWs to support	15	Moderate methodological limitations (the authors do not consider potential bias and analysis methods)	NA	NA	No concerns about adequacy of data (the study provides rich data)	NA	NA
58	Conflicting information	21	Moderate methodological limitations (the authors do not explain potential bias and sampling methods)	NA	NA	Moderate concerns (the study offers thin data)	NA	NA
Facilitators								
Facilitators to Recommended Breastfeeding								
Key Theme: *Perceptions*								
59	Breastfeeding is a cheap and hygienic way of feeding	1; 2; 4; 7; 9; 13	Moderate methodological limitations (all studies with moderate methodological limitations)	Minor concerns about relevance (the review fining is relevant in six countries: Dominican Republic, Tanzania, Ghana, Vietnam, Lao PDR and Cambodia)	Minor concerns about coherence (data consistent within and across both studies)	Minor concerns about adequacy (four studies provide rich data, but two studies show only thin data)	High confidence	This finding was graded as high confidence because of minor concerns about relevance, coherence and adequacy; and moderate methodological limitations.
60	Breastfeeding relieves uncomfortable fullness of breasts	4	Moderate methodological limitations (the study does not state analysis methods and potential bias)	NA	NA	Moderate concerns about adequacy (the authors provide only thin data)	NA	NA
61	Breastfeeding strengthens the bond between the mother and child	5; 9	Moderate methodological limitations (both studies do not consider potential bias. One does not state analysis methods. The other one does not describe sampling)	Moderate concerns about relevance (partial relevance as the studies were from only two settings: Colombia and Laos PDR)	Minor concerns about coherence (data consistent within and across both studies)	Moderate concerns about adequacy (one study provides only thin data. The other one provides rich data)	Moderate confidence	This finding was graded as moderate confidence because of moderate concerns regarding methodology, relevance and adequacy and minor concerns about coherence.
Key Theme: *Healthcare Services*								
62	Mothers and family members received advice and encouragement at healthcare setting(s) (e.g., prenatal visits, delivery, health posts, health worker home visits)	3; 10; 13; 16; 17; 18	Moderate methodological limitations (five studies with moderate methodological limitations, one study with minor limitations)	Minor concerns about relevance (the review fining is relevant in six countries: Ghana, Haiti, Cambodia, Ethiopia, Zambia and Nigeria)	Minor concerns about coherence (data consistent within and across both studies)	Substantial concerns about adequacy (four studies show poor data. Two studies show moderately rich data)	Moderate confidence	This finding was graded as moderate confidence because of substantial concerns about adequacy, moderate methodological limitations, minor concerns about relevance and coherence.
63	Mothers and family members receive advice and counseling from HWs	2; 3; 4; 9; 13; 14; 15; 16; 17; 18; 19	Moderate methodological limitations (ten studies with moderate methodological limitations, one study with minor limitations)	Minor concerns about relevance (the review fining is universal in six countries: Tanzania, Ghana, Lao PDR, Cambodia, Democratic Republic of Congo, Ethiopia, Zambia, Nigeria and Malawi)	Minor concerns about coherence (data consistent within and across both studies)	Substantial concerns about adequacy (four studies provide moderately rich data. Other studies offer only thin data)	Moderate confidence	This finding was graded as moderate confidence because of substantial concerns about adequacy, moderate methodological limitations, minor concerns about relevance and coherence.
Key Theme: *Infant-Specific Factors*								
64	Crying provides guidance for baby feeding practices	2; 10	Minor methodological limitations (one study does not state sampling methods and potential bias)	Moderate concerns about relevance (partial relevance as the studies were from only two settings: Tanzania and Haiti)	Minor concerns about coherence (data consistent within and across both studies)	Minor concerns about adequacy (both studies provide rich data overall)	High confidence	This finding was graded as high confidence due to only minor concerns about adequacy, relevance and methodological limitations and moderate concerns about relevance.
Key Theme: *Mother-Specific Factors*								
65	Mothers understand the importance of breastfeeding	1; 2; 3; 4; 5; 7; 9; 10; 11; 15; 16; 17; 18; 19; 21; 22	Moderate methodological limitations (14 studies with moderate methodological limitations, two studies with minor limitations)	Minor concerns about relevance (the review fining is universal in 12 countries: Dominican Republic, Tanzania, Ghana, Colombia, Vietnam, Lao PDR, Haiti, Ethiopia, Zambia, Nigeria, Malawi and Kenya)	Minor concerns about coherence (data consistent within and across both studies)	Minor concerns about adequacy (the majority of studies provide rich information while three studies offer only thin data)	High confidence	This finding was graded as high confidence because of minor concerns about relevance, coherence and adequacy; and moderate methodological limitations.
66	Mothers understand the importance of colostrum	2; 7; 14; 19	Moderate methodological limitations (all studies with moderate methodological limitations)	Minor concerns about relevance (the review finding is relevant in four countries: Tanzania, Vietnam, Democratic Republic of Congo and Malawi)	Minor concerns about coherence (data consistent within and across both studies)	Minor concerns about adequacy (all studies provide moderately rich data overall)	High confidence	This finding was graded as high confidence because of minor concerns about relevance, coherence and adequacy; and moderate methodological limitations.
Key Theme: *Social and Cultural Factors*								
67	Cultures and ethnicity facilitate early initiation of breastfeeding	3	Moderate methodological limitations (the authors do not explain analysis methods and potential bias)	NA	NA	No concerns about adequacy (the study provides rich data)	NA	NA
68	Family support promotes breastfeeding	1; 2; 7; 9	Moderate methodological limitations (all studies with moderate methodological limitations)	Minor concerns about relevance (the review finding is relevant in four countries: Dominican Republic, Tanzania, Vietnam and Lao PDR)	Minor concerns about coherence (data consistent within and across both studies)	Substantial concerns about adequacy (only one study provides rich data. Other studies show poor data)	Moderate confidence	This finding was graded as moderate confidence because of moderate methodological limitations, minor concerns regarding relevance and coherence and substantial concerns about adequacy.
69	Praise and affirmation from a partner and/or family facilitate breastfeeding	2	Moderate methodological limitations (the authors do not explain sampling and potential bias)	NA	NA	No concerns about adequacy (the study provides rich data)	NA	NA
Facilitators to Complementary Feeding								
Key Theme: *Food Security*								
70	Some foods are available	16; 18; 22	Moderate methodological limitations (all studies with moderate methodological limitations)	Moderate concerns about relevance (partial relevance as the studies are from only three settings: Ethiopia, Nigeria and Ghana)	Minor concerns about coherence (data consistent within and across both studies)	Minor concerns about adequacy (all studies provide moderately rich data overall)	Moderate confidence	This finding was graded as moderate confidence because of moderate concerns about methodology and relevance; and minor concerns about coherence and adequacy.
Key Theme: *Social Support*								
71	Family support promotes complementary feeding	6; 15; 16; 21	Moderate methodological limitations (all studies with moderate methodological limitations)	Moderate concerns about relevance (partial relevance as the studies are from only three countries: Bangladesh, Ethiopia and Kenya)	Minor concerns about coherence (data consistent within and across both studies)	Minor concerns about adequacy (three studies provide rich data, but one study offers only thin data)	Moderate confidence	This finding was graded as moderate confidence because of minor concerns about coherence and adequacy; and moderate concerns regarding relevance and methodology.
Key Theme: *Mother-Specific Factors*								
72	Mothers understand the importance of complementary feeding	15; 16; 18	Moderate methodological limitations (all studies with moderate methodological limitations)	Moderate concerns about relevance (partial relevance as the studies are from only two countries, Ethiopia and Nigeria)	Minor concerns about coherence (data consistent within and across both studies)	Minor concerns about adequacy (all studies provide rich data)	Moderate confidence	This finding was graded as moderate confidence because of minor concerns about coherence and adequacy; and moderate concerns
Key Theme: *Healthcare Services*								
73	Mothers receive IYCF advice and counseling at healthcare facilities	15; 18; 19; 21	Moderate methodological limitations (all studies with moderate methodological limitations)	Minor concerns about relevance (the review findings is relevant in four countries: Ethiopia, Nigeria, Malawi and Kenya)	Minor concerns about coherence (data consistent within and across both studies)	Substantial concerns about adequacy (three studies provide thin data. Only one study shows moderately rich data)	Moderate confidence	This finding was graded as moderate confidence because of minor concerns about coherence and adequacy; and moderate methodological limitations and substantial concerns about adequacy.

NA: refers to nothing.
