# Supplementary material for: Qualitative Studies of Infant and Young Child Feeding in Lower-Income Countries: A Systematic Review and Synthesis of Dietary Patterns
Source: Nutrients. 2017 Oct 18;9(10):1140. doi: 10.3390/nu9101140 (PMC5691756; doi:10.3390/nu9101140)
Supplement: Supplementary file 1 [file nutrients-09-01140-s001.pdf]

Table S1. ENTREQ checklist (Enhancing transparency in reporting the synthesis of qualitative research) *.

No. Item	Guide Questions/Description	Reported on Page
1. Aim	To identify barriers and facilitators to achieving recommended feeding practices by parents and families in low income countries.	Title & 5–6
2. Synthesis methodology	Identify the synthesis methodology or theoretical framework which underpins the synthesis, and describe the rationale for choice of methodology (e.g. meta-ethnography, thematic synthesis, critical interpretive synthesis, grounded theory synthesis, realist synthesis, meta-aggregation, meta-study, framework synthesis)	8–9
3. Approach to searching	The search was pre-planned. Comprehensive search strategies were undertaken to seek all available studies.	6–7
4. Inclusion criteria	Included studies used widely accepted qualitative data collection methods, with well-described methodology, including for example: interviews, focus groups, direct observation, and participatory action research. Included studies also needed to have provided a clear description of recognized qualitative data analysis methods (e.g., grounded theory, narrative analysis, content analysis, thematic analysis). Excluded studies included those for which it was difficult to extract qualitative data, e.g., mixed methods studies without clearly labeled data, or studies in settings where perceptions of parents or caregivers around infant and young child feeding could not be clearly identified, such as summaries or aggregated data. Commentaries, protocols, and systematic reviews were not included in the analysis. Additionally, as the focus was on research from resource limited settings, studies from countries other than those defined by the World Bank as low-income countries and lower-middle income countries (which have a Gross National Income per capita of less than $4,125) were excluded.	6
5. Data sources	The following electronic databases were considered to be the most relevant for the topic and were searched: MEDLINE (PubMed); Embase; Cumulative Index to Nursing and Allied Health Literature (CINAHL: EBSCOhost). The included gray literature was initially identified through listing of relevant websites to search for organizations working in nutrition in lower-income countries (in consultation with experts working in the field who use and disseminate data through websites for related nutrition research). All data were limited to publications in English from the last 10 years (from 2006 to 2016).	6–7
6. Electronic Search strategy	Appendix A describes the literature search.	Appendix A
7. Study screening methods	Two independent reviewers screened study titles and abstracts for suitability against inclusion and exclusion criteria. The decision to include or exclude a study was required to be agreed on by both reviewers. If after consultation a decision wasn't reached by the two reviewers, a third reviewer made the final decision.	9–12

8. Study characteristics	Table 1 presents the characteristics of the included studies (author(s), year of publication, country, population, number of participants, data collection, methodology, analysis and limitation of the studies).	Table 1
9. Study selection results	A flow diagram using PRISMA guidelines for reporting of systematic reviews is presented in Figure 1 in reporting of the selection process and results. We identified 539 studies after removing duplicates and excluded 512 studies for the following reasons: 1) studies were not from low-middle income countries; 2) studies were related to program evaluation and 3) the target populations included HIV positive mothers and caregivers. In addition, we excluded four studies because there was no text available. As a result, we included 21 studies in qualitative synthesis.	Figure 1, Table 1
10. Rationale for appraisal	Critical Appraisal Skills Program (CASP) guidelines and GRADE-CERQual were used to assess qualitative evidence syntheses findings. CASP offers a valid checklist to help researchers appraise and understand qualitative studies better. GRADE-CERQual helps researchers assess how much confidence to place in findings from a qualitative evidence synthesis.<br>First,each selected document was initially assessed for quality and internal validity according to the CASP checklist for qualitative research. The CASP checklist includes 10 questions to appraise the quality of qualitative research. These assessments for each study can be seen in the final column of Table 1 with reference to the CASP appraisal question number where the study presented potential quality limitations. Selected studies met minimum criteria defined through the checklist including domains such as appropriateness of study design, data collection techniques, and analysis methods used. At the second level of appraisal, the GRADE-CERQual guidance was used to differentiate emergent findings strongly supported or less well supported. Two reviewers (AB and AK) independently reviewed studies using guidance derived from GRADE-CERQual to reach consensus of the quality of findings emergent from included studies. The results of this appraisal are presented in Table 2.	7–8
11. Appraisal items	CASP guidelines and GRADE-CERQual were used to assess qualitative evidence syntheses findings.	8
12. Apprais l process	Appraisal was conducted independently by two independent reviewers. The two reviewers discussed if consensus was required.	8
13. Appraisal results	Appraisal results are presented in Tables 1 and 2.	Tables 1–2

14. Data extraction	For organization of extracted data, a unified matrix was utilized to record specific characteristics of included studies. Extracted data included: reference details (author, year, title, journal/publisher); country/region of study; objectives or aims of the study; study design including methodological approaches (e.g., interviews/focus groups) and conceptual basis underlying the study (e.g., Grounded Theory); analysis method(s); sampling methodology and sample size; and initial assessment of the methodological limitations of the study. The initial results of the selection process and data abstraction are presented in Table 1.<br>Additional steps were taken in the data extraction phase that involved expanding the matrix (Table 1) to include participant characteristics, summaries of key outcomes/results reported, and the emergent review findings for which the study contributes evidence. These details are given in Appendix B.	7
15. Software	Nvivo 11 qualitative software was used.	7
16. Number of reviewers	Two reviewers were involved in coding and analysis.	8
17. Coding	Thematic analysis was employed to identify domains descriptive of the data for investigation and presentation. This thematic analysis led to a more interpretive phase in order to understand how the themes identified may represent barriers and facilitators to change infant and young child feeding.	8
18. Study comparison	Similar findings were coded into key themes and categories within and across studies. GRADE-CERQual was used to assess confidence in qualitative evidence syntheses findings across studies.	Table 2
19. Derivation of themes	The process of deriving the themes and categories were inductive to assess previously researched phenomena.	8
20. Quotations	Appendix B provides findings and quotations from the primary studies to illustrate themes and constructs, and identify whether the quotations were participant quotations of the author's interpretation.	Appendix B
21. Synthesis output	Synthesis output is presented in Table 3. Four categories of barriers to recommended breastfeeding practices were identified, and three categories of barriers to recommended complementary feeding practices were identified. Barriers to breastfeeding included factors specific to infant or mother, and cross-cutting beliefs and perceptions, as well as a pervasive lack of support for breastfeeding, from families, health workers and due to time poverty. Several categories of facilitators were also identified from the literature reviewed, including food security, social support, and individual infant and maternal factors. The review focused on identifying studies that related the experiences and first-hand accounts of family members responsible for providing for the care and nutritional needs of young children under 2 years of age. Through this systematic qualitative review and synthesis, hypothesized barriers and facilitators to improving infant and young child feeding were identified. The findings presented in this review are directly applicable to social and behavioral change initiatives in low resource settings aimed at improving practices for better health and nutrition of young children.	Table 3

* Reference: Tong A, Flemming K, McInnes E, Oliver SA, Craig J. Enhancing transparency in reporting the synthesis of qualitative research: ENTREQ. BMC Medical Research Methodology 2012, 12:181.
